# Differentiation of otitis media-causing bacteria and biofilms *via* Raman spectroscopy and optical coherence tomography

**DOI:** 10.3389/fcimb.2022.869761

**Published:** 2022-08-10

**Authors:** Andrea K. Locke, Farzana R. Zaki, Sean T. Fitzgerald, Kavya Sudhir, Guillermo L. Monroy, Honggu Choi, Jungeun Won, Anita Mahadevan-Jansen, Stephen A. Boppart

**Affiliations:** ^1^ Vanderbilt Biophotonics Center, School of Engineering, Vanderbilt University, Nashville, TN, United States; ^2^ Department of Biomedical Engineering, School of Engineering, Vanderbilt University, Nashville, TN, United States; ^3^ Department of Chemistry, College of Arts and Science, Vanderbilt University, Nashville, TN, United States; ^4^ Beckman Institute for Advanced Science and Technology, University of Illinois at Urbana–Champaign, Urbana, IL, United States; ^5^ Department of Bioengineering, The Grainger College of Engineering, University of Illinois at Urbana–Champaign, Urbana, IL, United States; ^6^ Department of Otolaryngology - Head & Neck Surgery, Vanderbilt University Medical Center, Nashville, TN, United States; ^7^ Department of Surgery, Vanderbilt University Medical Center, Nashville, TN, United States; ^8^ Department of Neurological Surgery, Vanderbilt University Medical Center, Nashville, TN, United States; ^9^ Carle Illinois College of Medicine, University of Illinois at Urbana–Champaign, Urbana, IL, United States; ^10^ Department of Electrical and Computer Engineering, University of Illinois Urbana-Champaign, Urbana, IL, United States

**Keywords:** bacteria, optical coherence tomography, Raman spectroscopy, biofilms, otitis media, biophotonics, optical spectroscopy, bacterial infection

## Abstract

In the management of otitis media (OM), identification of causative bacterial pathogens and knowledge of their biofilm formation can provide more targeted treatment approaches. Current clinical diagnostic methods rely on the visualization of the tympanic membrane and lack real-time assessment of the causative pathogen(s) and the nature of any biofilm that may reside behind the membrane and within the middle ear cavity. In recent years, optical coherence tomography (OCT) has been demonstrated as an improved *in vivo* diagnostic tool for visualization and morphological characterization of OM biofilms and middle ear effusions; but lacks specificity about the causative bacterial species. This study proposes the combination of OCT and Raman spectroscopy (RS) to examine differences in the refractive index, optical attenuation, and biochemical composition of *Haemophilus influenzae*, *Streptococcus pneumoniae*, *Moraxella catarrhalis*, and *Pseudomonas aeruginosa*; four of the leading otopathogens in OM. This combination provides a dual optical approach for identifying and differentiating OM-causing bacterial species under three different *in vitro* growth environments (i.e., agar-grown colonies, planktonic cells from liquid cultures, and biofilms). This study showed that RS was able to identify key biochemical variations to differentiate all four OM-causing bacteria. Additionally, biochemical spectral changes (RS) and differences in the mean attenuation coefficient (OCT) were able to distinguish the growth environment for each bacterial species.

## Introduction

Otitis media (OM), an inflammatory disease of the middle ear, is one of the most common diagnoses for pediatric patients and often follows upper respiratory tract infections (Danishyar and Ashurst, 2017). It is one of the leading causes of outpatient antibiotic use for children (Palms et al., 2019), resulting in more than 25 million physician visits and $4.3 billion in public healthcare costs, annually, in the United States alone (Tong et al., 2018). Approximately 80-90% of children present with some form of OM by the age of three. Thus, an appropriate diagnosis and treatment regimen is crucial to minimize the risk of delayed speech development and socialization skills that may result from the long-term reduction of auditory acuity during chronic states of the disease. OM involves the accumulation of a middle ear effusion (MEE) caused by Eustachian tube dysfunction. In the absence of acute infection, this condition is termed OM with effusion (OME). However, planktonic bacteria from the upper respiratory tract can migrate into the middle ear through the Eustachian tube and colonize within the nutrient-rich MEE. This migration may lead to the onset of an acute OM (AOM) infection by one or more pathogens that presents with physical symptoms (e.g., otalgia, otorrhea, fever, irritability, restlessness, and poor feeding) and tympanic membrane (TM) bulging or erythema in children. Cases of OME can resolve spontaneously within months, and many AOM cases typically resolve within 1-2 weeks with appropriate administration of antibiotics. However, identifying and appropriately treating a single persistent/chronic or multiple recurrent infections is still an ongoing challenge as most of these cases do not respond to antibiotic therapy and surgical intervention is advised (Hoberman et al., 2011).

Evidence has pointed towards the importance of biofilm formation in the occurrence and persistence of chronic OM. Bacterial biofilms are self-assembling microenvironments that provide a protective habitat for microorganisms to colonize and grow. The biopolymeric matrix adheres to tissue structures and protects the pathogens from external agents like host immune cells or antimicrobial drugs. Researchers have reported findings up to 92% of middle ear mucosal biopsies sampled from patients with chronic OM showed the presence of biofilms (Kaya et al., 2013; Gu et al., 2014). OM-related biofilms usually contain one or multiple species of the primary otopathogenic bacteria, including: *Streptococcus pneumoniae*, non-typeable *Haemophilus influenzae* (NTHI), *Moraxella catarrhalis*, *Pseudomonas aeruginosa*, and *Staphylococcus aureus* (Fergie et al., 2004; Thornton et al., 2011). Although targeted therapies like species-specific antibiotic agents can be more effective than broad-spectrum antibiotics when pathogens are in free-floating (i.e., planktonic) form, biofilms exhibit unique antibiotic resistance mechanisms that affect drug treatment efficacy (Sharma et al., 2019). Therefore, detection and characterization of infection in a planktonic or biofilm state are critical diagnostic criteria needed to monitor the progression of chronic forms of OM. Consideration of these aspects of OM pathology would not only improve procedures for prescribing antibiotic therapies, but allow for tracking of drug treatment response in chronic OM.

Another challenge in the administration of treatment is identifying the cause of OM. Although a large number of AOM cases are associated with bacterial pathogens, in approximately 5-30% of AOM cases in children, the causative pathogen is viral or a combination of both (Shoichi et al., 2019). With viral infections, the administration of antibiotics is unnecessary, or in combined viral/bacterial infections, monitoring of antibiotic response over a longer period of time may be needed. This is because the bacterial infection could have resolved, but symptoms relating to a viral presence persisted. There are also instances where the presence of an effusion in the middle ear is serous (i.e., OME) without signs of infection (Vanneste and Page, 2019). In these cases, antibiotics treatments are not recommended under current guidelines, and “watchful waiting” is recommended (Lambert, 2016). Therefore, an OM diagnostic tool capable of real-time identification of bacterial presence and pathogen speciation is needed to help guide clinical decision making for the most appropriate treatment regimen.

Visual or digital otoscopy is the current clinical standard for the diagnosis of OM, but can be subjective amongst practitioners. Alternative methods like pneumatic otoscopy or tympanometry examine the motility of the TM in response to pressure oscillations in the ear canal to determine the presence of MEE, but lack the specificity of pathogen identification and the ability to identify or track the evolution of the effusion or biofilm over the course of treatment and progression/regression of the disease. Tympanocentesis, a minor surgical procedure to aspirate a MEE from the middle ear cavity with a small gauge needle, remains the gold standard for typing of OM-causing bacterial species, but is only used in complex cases that do not respond to first-line antibiotics like amoxicillin or penicillin. However, in some cases (~40%) the extracted middle ear fluid is culture-negative, and more time consuming and complex tools like polymerase chain reaction (PCR) and DNA sequencing are needed to determine speciation (Xu et al., 2011). These limitations imposed by current diagnostic methods have resulted in treatment guidelines for OM recommending the use of broad-spectrum antibiotics that often adds to the risk of antibiotic resistance (Center for Disease Control and Prevention, 2019). This risk includes increases in the incidence of adverse effects such as disruption of the gut microbiome (Francino, 2016), upset stomach, or diarrhea (Gerber et al., 2017), which can lead to other poor health outcomes and diseases (Holmes et al., 2011; Chen et al., 2021). Thus, advances in more specific and less labor-intensive diagnostic tools are essential to improve clinical treatment decisions and patient outcomes.

Building off of existing visual and digital otoscopy platforms, optical methods have garnered interest in OM diagnostics as they are inherently non-invasive, fast, can be readily applied *in vivo*, and offer a wide range of compositional or structural information about microbes and the microenvironment they inhabit. Optical coherence tomography (OCT) is an interferometric imaging modality that provides micron-scale resolution of tissue morphology. This optical modality has been investigated for application in OM to detect the presence of middle ear effusions and biofilms (Nguyen et al., 2012; Monroy et al., 2015; Won et al., 2021b), correlate TM thickness to a chronic state of the disease (Dsouza et al., 2018), and study the longitudinal effects of antibiotic treatment on biofilm growth and MEEs (Won et al., 2021a). To date, OCT is the only technique that can provide direct visualization of biofilms adherent on the TM *in vivo*. Raman spectroscopy (RS) is a vibrational spectroscopic modality that excels in its ability to probe molecular composition through the inelastic scattering of light. It has previously been reported for use in OM diagnostics by identifying causative bacterial species, grown on agar *in vitro*, with 97% classification accuracy (Ayala et al., 2017). Also, RS has been used to characterize compositional differences in non-infected MEEs *in vitro*, where thicker mucoid MEE could be discriminated from thinner serous effusions with 92% accuracy (Pandey et al., 2018). This is significant because mucoid MEEs are less easily cleared by middle ear ciliary action and are associated with more chronic episodes of OM (Matković et al., 2007). In a recent study, a combined Raman coupled low-coherence interferometry (LCI) probe was developed for the real-time detection and differentiation of microbial pathogens (i.e., *Pseudomonas aeruginosa* and *Streptococcus pneumoniae*) in the middle ear (Zhao et al., 2016). Therein, RS was employed to identify the planktonic bacterial species, and LCI was utilized to determine the depth-resolved structural information from the TM and to visually guide the placement of the RS probing beam. Thus, this work demonstrated the advantages of combining spectroscopy with imaging to provide a more informed description of the nature of bacterial infection within the middle ear.

The two modalities, OCT and RS, offer very different but complementary information about effusion scattering properties and biofilm microstructure, and cellular biochemical composition, respectively. While OCT can identify MEEs and biofilm formation, track film thickness, and investigate optical properties like refractive index, attenuation, or other scattering properties (Tearney et al., 1995; Balaev et al., 2003; Chang and Bowden, 2019), it lacks chemical specificity which could be used to determine which species are responsible for any persistent disease like chronic OM. On the other hand, RS provides a comprehensive biomolecular identification of the bacterial, biofilm, and MEE, but cannot provide information about the structural organization of functional characteristics of tissue when applied *in vivo*. Thus, there is a growing interest in exploring a combined multimodal optical system that utilizes the advantages of both techniques. In the context of acute and chronic OM, a multimodal RS-OCT platform would be able to detect MEE and biofilm formation and identify the causative bacterial pathogen(s) to better inform the clinical guidance of appropriate treatment, and allow for longitudinal observation of a patient’s response to the antibiotic therapy.

The goal of this study is to characterize OM-causative bacterial species, grown in different *in vitro* environments, through the independent techniques of RS and OCT. This will facilitate an understanding of the key biochemical and structural differences between bacteria found on agar, in a fluidic environment (i.e., planktonic), and within established biofilms. In particular to OM, this study aims to identify discriminatory markers that can be used to distinguish the four most common causative bacterial species: *Haemophilus influenzae*, *Streptococcus pneumoniae*, *Moraxella catarrhalis*, and *Pseudomonas aeruginosa*.

## Results

To characterize differences between OM-causing bacterial species when grown as colonies on brain heart infusion (BHI) agar, in a fluidic environment, or a biofilm matrix, RS and OCT measurements were acquired from four of the most common otopathogenic OM bacterial species: *H. influenzae*, *S. pneumoniae*, *M. catarrhalis*, and *P. aeruginosa* under all three growth conditions.

### Raman characterization of OM-causing bacteria in different microbial growth environments

**Figure 1:**
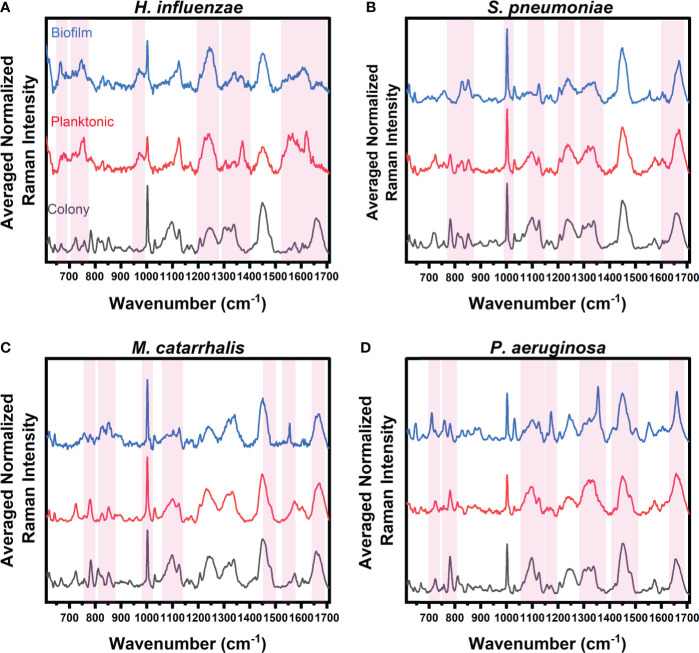
Average Raman spectra of **(A)** *H*. *influenzae*, **(B)** *S. pneumoniae*, **(C)** *M. catarrhalis*, and **(D)** *P. aeruginosa* comparing key Raman spectral differences relating to bacterial growth under three different environments (grey: colony on agar, red: planktonic form, and blue: biofilms on CaF_2_ substrates). The red bands highlight the wavenumbers with SMLR weighted features contributing ≥60% in differentiating each species’ growth forms.

Raman spectra were acquired using a benchtop inVia™ confocal Raman microscope (Renishaw, United Kingdom) fitted with a 785 nm excitation light. Raman spectra from colonies grown on brain heart infusion (BHI) agar, liquid cultures, and biofilm samples were acquired in triplicate, processed for noise smoothing and fluorescence background subtraction, then averaged before analysis. Sparse Multinomial Logistic Regression (SMLR), a supervised classification model, was used to analyze the Raman spectra by only selecting spectral bands that were most important to species/growth form differentiation. By simultaneously performing both feature selection and classification, the SMLR model demonstrated the ability for RS to discriminate between the experimental groups as well as identify which Raman biochemical components were adept at accurate differentiation. The SMLR classification model was trained using spectra from each bacterial species in colonies, planktonic cells, and biofilm samples to determine important spectral differences. The penalty term, lambda (λ), was set to 1 and represented a high degree of sparsity (i.e., the degree by which the set of features are reduced to important weighted features). The k-fold cross-validation was set to 5, which allowed the data to be randomly divided into k-number of groups to retrain and ensure that the model was not overfitted to any particular subset of data. **Figure 1** highlights the key Raman spectral bands identified by the SMLR model, that contribute to the differentiation of each growth environment with a threshold weighted percentage of ≥60%.

The Raman spectra from the three forms of bacterial growth environments of *H. influenzae* show distinct Raman differences, as seen in **Figure 1A**. The colony, planktonic (pelleted) cells (i.e., planktonic form), and biofilm forms display changes in the Amide I protein structural bonds between 1643 and 1650 cm^-1^. Also, the Raman spectra of the biofilms show a relative intensity increase as shown in the broad Raman band at ~1610 cm^-1^ (cytosine: NH_2_). Additional intensity increase is also observed at ~663 cm^-1^ (Guanine and tyrosine) for the biofilms of *H. influenzae* compared to the other growth forms. **Figure 1A** also highlights differences in the Raman spectra of the planktonic form of *H. influenzae.* Of note is the reduction in the Raman peak intensity at ~1342 cm^-1^ (Guanine: DNA/RNA; CH deformation of proteins and carbohydrates) in comparison to its colony and biofilm forms.

**Figure 1B** highlights the main Raman spectral features of *S. pneumoniae* that contribute to bacterial differentiation in the three different growth environments. These features include a shift in the Amide I band from 1660 cm^-1^ (C=C ***cis***, lipids, structural proteins) to 1670 cm^-1^ (C=C ***trans***, lipids) for bacteria residing in colonies compared to those in both planktonic and biofilm forms. A reduction in the intensities of the Raman bands relating to Guanine at 1320 and ~1357 cm^-1^ is also seen in planktonic and biofilm forms compared to the bacterial colonies. Also shown is a reduction in the relative Raman intensity of asymmetric phosphate stretching modes at 1230 cm^-1^ and phosphodioxy groups in nucleic acids of the biofilms, compared to the signal intensity of the agar colonies.

Spectral band differences from *M. catarrhalis* in colonies, and planktonic and biofilm forms are shown in **Figure 1C**. Compared to both agar colonies and planktonic form, biofilms of *M. catarrhalis* demonstrated a reduction in relative Raman intensity at 1098 cm^-1^ (C-N in lipids) but maintained a relatively similar Raman intensity at 1124 cm^-1^ (C-C skeletal acyl backbone in lipids). Also seen were a reduction in the relative intensity at ~780 cm^-1^ (uracil/cytosine ring breathing) and the presence of a sharp Raman peak at ~1554 cm^-1^ (tryptophan and exopolysaccharide) for the biofilms compared to the other forms. In differentiating the agar colonies from the other environments, the Amide I band was observed at 1660 cm^-1^ (C=C ***cis***, lipids, fatty acids, structural proteins) while the corresponding peak for planktonic and biofilm forms resided at 1670 cm^-1^ (C=C ***trans***, lipids, fatty acids).

Raman spectroscopy was also able to differentiate amongst *P. aeruginosa* in the three different growth environments, as highlighted in **Figure 1D**. Raman peaks relating to pyocyanin secretion in the biofilm environment at 1174 and 1355 cm^-1^ were significant in differentiating the biofilms from both planktonic and colony forms. Other key differences included the presence of a strong band at 1315 cm^-1^ relating to the CH_3_CH_2_ twisting in lipids, and a shift of the 1675 cm^-1^ Raman peak relating to the β-sheet of Amide I vibrational bonds of the biofilms, when compared to the agar colony and planktonic forms. Also, the important key features to differentiate bacterial agar colonies from the other growth forms are observed to be differences in Raman bands at 1098 cm^-1^ (Phosphodioxy group in nucleic acids), 1328 cm^-1^ (CH_3_CH_2_ wagging in purine bases of nucleic acids), 1360 cm^-1^ (tryptophan), 1456 cm^-1^ (DNA), and between 1675 and 1700 cm^-1^ (Amide I disordered structure).

Based on these key changes among growth environments across each bacterium, the SMLR model demonstrated the ability to accurately differentiate bacterial forms with a minimum sensitivity of 95% and specificity of 98%, as summarized in **Table 1**.

**Table 1:** A summary of the sensitivity and specificity for SMLR differentiation of three different growth environments for individual bacterial species using RS.

*Bacterial Species*	Colony	Planktonic	Biofilm
*H. influenzae*			
Sensitivity (%) Specificity (%)	100 100	95.2 100	100 98.7
*S. pneumoniae*			
Sensitivity (%) Specificity (%)	100 98.5	100 100	96.7 100
*M. catarrhalis*			
Sensitivity (%) Specificity (%)	100 100	100 100	100 100
*P. aeruginosa*			
Sensitivity (%) Specificity (%)	100 98.0	100 100	96.7 100

#### Raman characterization and comparison across bacterial species

To determine the key Raman spectral features that would allow for differentiation amongst the four species, an SMLR model (λ = 1; k-fold = 5) was used to identify discriminatory factors in each growth environment.

**Figure 2:**
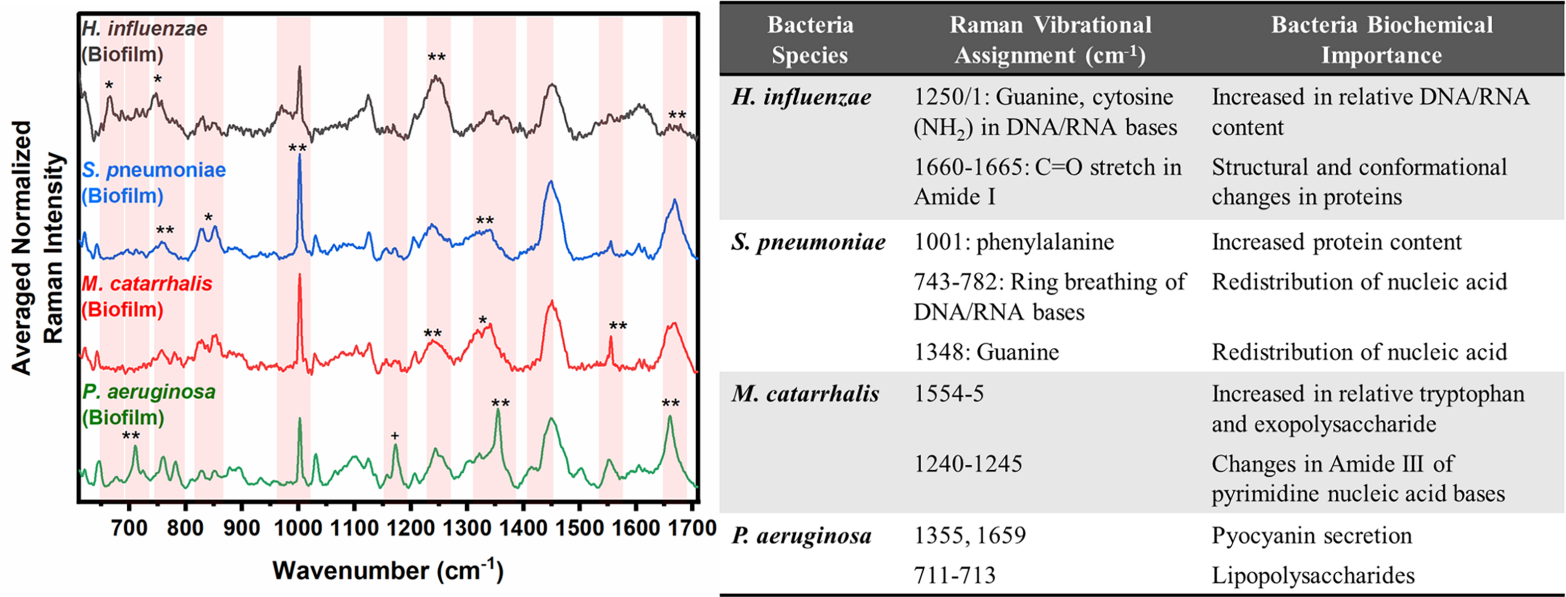
Averaged Raman spectra (left) highlighting key Raman features identified by SMLR to differentiate OM-causing bacterial biofilms. The table (right) provides a summary of the tentative biochemical assignments for the Raman peaks with 80% and above weighted feature contribution to the differentiation. The red bands highlight the wavenumbers with SMLR weighted features contributing to *60-79% and **≥80% in differentiating amongst the species’ biofilms.

In **Figure 2**, the key Raman bands with the highest weighted percentages for (**≥ 80%, *≥ 60%) biofilm differentiation between the four species are identified and highlighted. In differentiating each species from the other three bacteria, the SMLR model result in sensitivities and specificities greater than 92.3% for all four species (**Table 2**). Compared to all species, biofilms of *H. influenzae* showed a significant reduction in intensity of the Amide I Raman band at 1655-1665 cm^-1^. Raman spectra of *S. pneumoniae* biofilms show a redistribution of nucleic acid with a relative lower intensity in the 740-782 cm^-1^ Raman bands, and a relatively higher intensity in the 800-850 cm^-1^ region. *M. catarrhalis* exhibits increased relative Raman intensity at 1554 cm^-1^ relating to tryptophan and exopolysaccharide content (Ayala et al., 2017). On the other hand, the Raman spectra of *P. aeruginosa* biofilms show a strong Raman band at 1355 cm^-1^ and a narrow but strong band at 1659 cm^-1^ that is not evident in the other bacterial species and is likely the result of the secretion of pyocyanin, a known bi-product of *P. aeruginosa* biofilms (Bodelón et al., 2016; Žukovskaja et al., 2017; Turasan, 2020).

**Table 2:** A summary of the sensitivity and specificity using SMLR to differentiate the four bacterial species for a given environment using RS.

Environment	*H. influenzae*	*S. pneumoniae*	*M. catarrhalis*	*P. aeruginosa*
Colony				
Sensitivity (%) Specificity (%)	100 97.2	98.0 99.3	93.8 100	97.9 100
Planktonic				
Sensitivity (%) Specificity (%)	100 97.6	92.9 96.3	98.2 98.5	100 100
Biofilms				
Sensitivity (%) Specificity (%)	94.4 98.9	93.8 96.7	92.3 97.9	100 100

**Figure 3:**
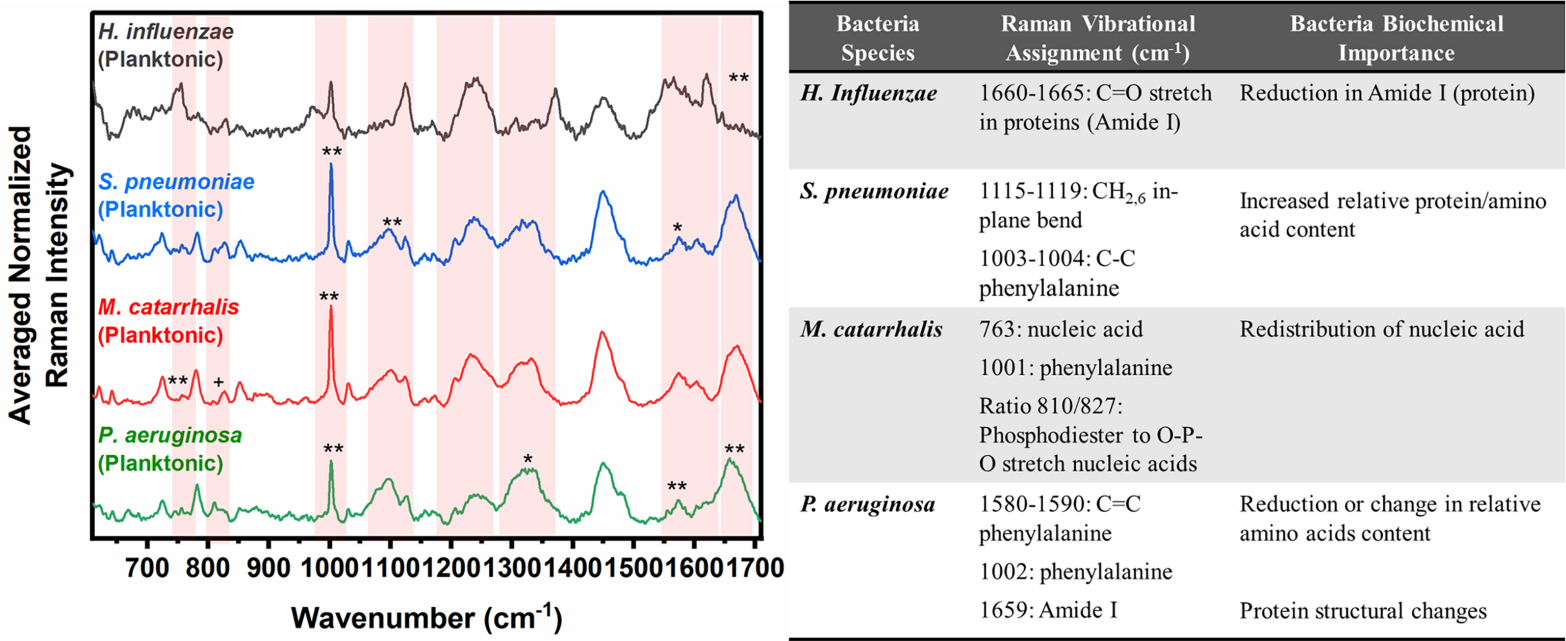
Averaged Raman spectra (left) highlighting key Raman features identified by SMLR to differentiate OM-causing bacteria planktonic form. The table (right) provides a summary of the tentative biochemical assignments of the Raman peaks. The red bands highlight the wavenumbers with SMLR weighted features contributing to *60-79% and **≥80% in differentiating amongst the species’ planktonic cells (*+spectral changes observed visually but not identified by the SMLR model*).

Herein, bacterial cells grown for 24 hours in liquid cultures were also measured to assess for any differences in the biochemical signatures of the cells grown as free-floating (i.e., planktonic) entities versus those grown on solid media or in a biofilm form. Prior to measurements, the free-floating cells were concentrated into a pellet to improve the signal-to-noise of the spectra. The key Raman peaks in differentiating each species from each other are highlighted in **Figure 3**. The corresponding SMLR model result in sensitivities and specificities greater than 92.9% in discriminating all four bacteria from each other (**Table 2**). The planktonic (pelleted) cells of *H. influenzae* show a significant redistribution of protein related content such as the Amide I Raman band between 1660-1670 cm^-1^ and phenylalanine at 1003 and 1104 cm^-1^. Spectral features of planktonic *S. pneumoniae* were difficult to differentiate from those of *M. catarrhalis*. However, compared to *S. pneumoniae*, *M. catarrhalis* exhibits a redistribution of nucleic acid content in the Raman spectra regions of 763 cm^-1^ and 810-830 cm^-1^. Raman spectra of planktonic *P. aeruginosa* show the absence of the Raman band at 1605 cm^-1^ (cytosine: NH_2_; ring C-C stretch of phenyl). Also, *P. aeruginosa* Amide I Raman band resides at 1659 cm^-1^, compared to *S. pneumoniae* and *M. catarrhalis*, both having the Amide I band residing at ~1670 cm^-1^.

**Figure 4:**
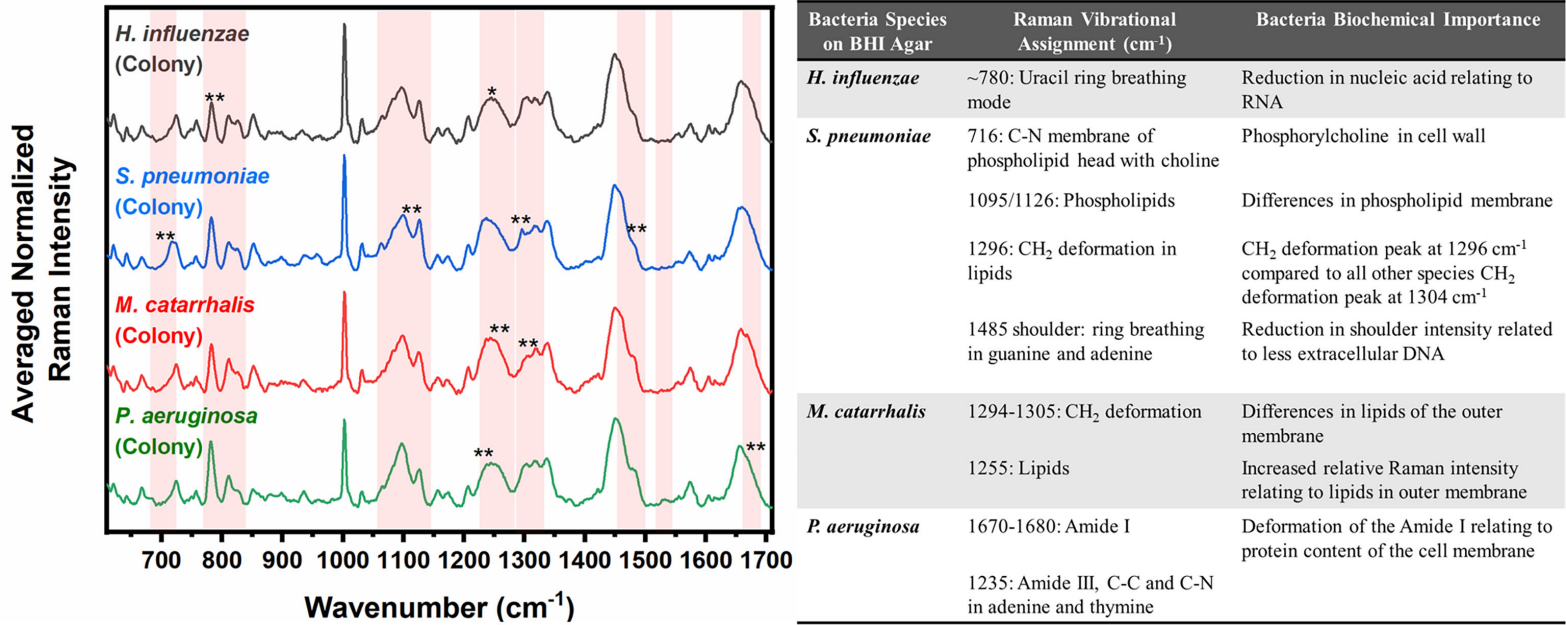
Averaged Raman spectra (left) highlighting key Raman features identified by SMLR to differentiate OM-causing bacterial colonies grown on BHI agar. The table (right) provides a summary of the tentative biochemical assignments of a few of the key Raman peaks. The red bands highlight the wavenumbers with SMLR weighted features contributing to *60-79% and **≥80% in differentiating amongst the species’ colonies.

Differences are also seen amongst the species of bacteria grown on BHI agar (**Figure 4**), and the resultant SMLR model show sensitivities and specificities above 93.8% in differentiating all four species from each other (**Table 2**). To note, compared to the others, the colonies of *H. influenzae* exhibit a reduction in Raman intensity at ~780 cm^-1^, relating to RNA content. A peak shift at ~716 cm^-1^ (phospholipids, choline group) is observed for *S. pneumoniae* colonies compared to the other species whose peak Raman band resides at 725 cm^-1^ (DNA). Another peak shift difference is observed at 1296 cm^-1^ relating to CH_2_ deformation in lipids, while the other bacterial species peak is located at ~1304 cm^-1^. The peak intensity ratio at I_1096_/I_1126_ for all four species also indicates differences (p< 0.001) in the phospholipid content across all four species with *S. pneumoniae* displaying the lowest ratio of 1.06 ± 0.11 compared to *M. catarrhalis* (1.36 ± 0.07), *H. influenzae* (1.29 ± 0.08), and *P. aeruginosa* (1.61 ± 0.11). *M. catarrhalis* shows a reduction in the lipid bands between 1295-1304 cm^-1^ and a significant increase (p< 0.001) in Amide III (1245 cm^-1^) Raman intensity; while the Amide I band of *P. aeruginosa* differs from the other species by a noticeable decrease in intensity at the shoulder of the 1670-1680 cm^-1^ Raman band.

### OCT characterization of OM-causing bacteria and their microbial environment

#### 2D and 3D OCT images of all forms

**Figure 5:**
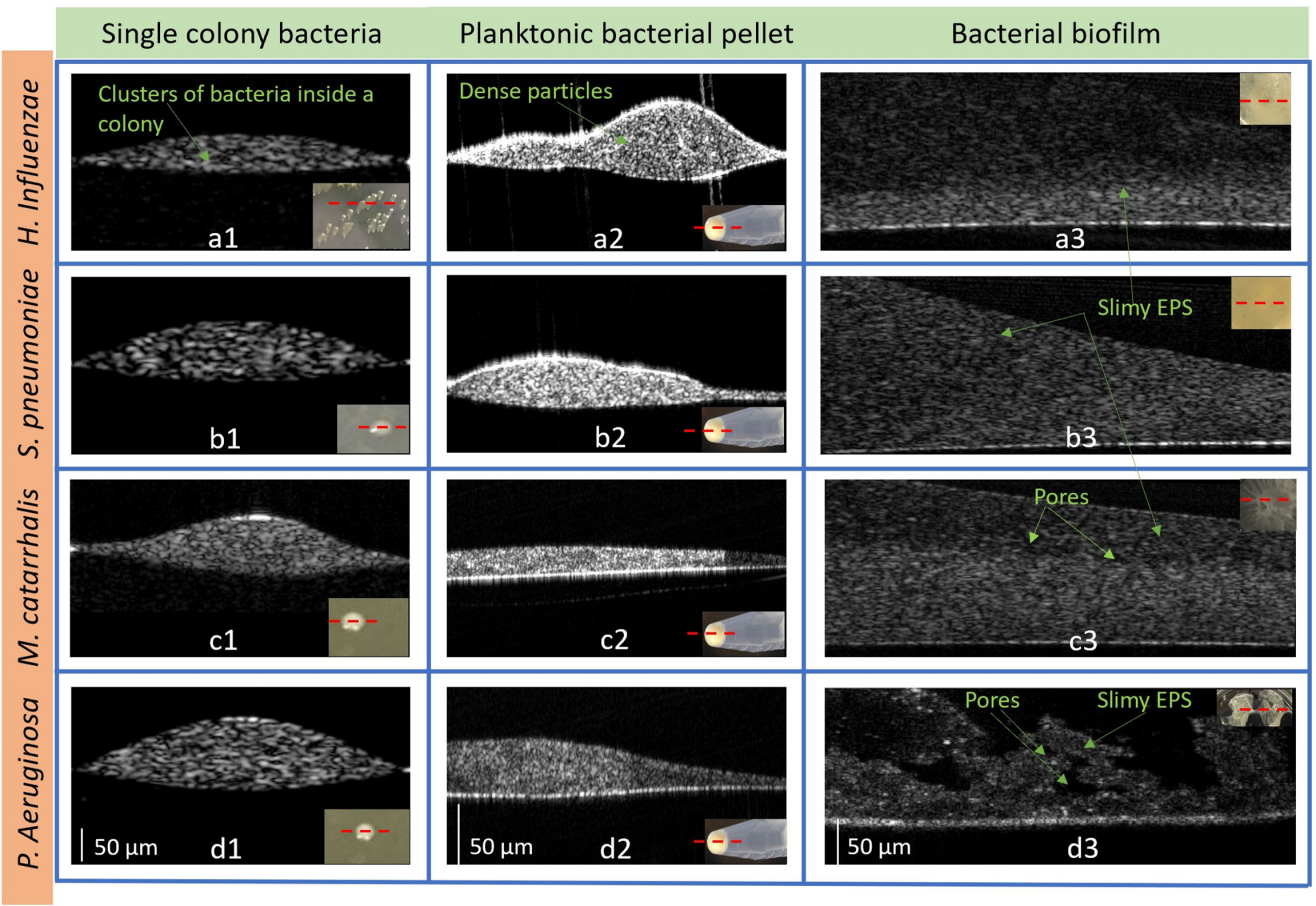
Representative 2D OCT images from *H. Influenzae* **(a1)** single-colony, **(a2)** planktonic (in pellet), and **(a3)** biofilm; *S. pneumoniae* **(b1)** single-colony, **(b2)** planktonic (in pellet), and **(b3)** biofilm; *M. catarrhalis* **(c1)** single-colony, **(c2)** planktonic (in pellet), and **(c3)** biofilm and *P. aeruginosa* in **(d1)** single-colony, **(d2)** planktonic (in pellet), and **(d3)** biofilm. The lower right insets show photos of the bacteria in each form. (**Supplementary Figure S3**: Representative 3D OCT images from *P. aeruginosa* are shown in **(a)** a single-colony, **(b)** planktonic (in pellet) and **(c)** biofilm and 3D videos of the three forms).

With a custom-built benchtop OCT system operating at a center wavelength of 1325 nm (**Supplementary Figure 1**), all four bacterial species were characterized and analyzed in colony, planktonic, and biofilm forms. **Figure 5** represents 2D cross-sectional OCT images from a single bacterial colony, planktonic bacterial pellet, and biofilm. The cross-sectional OCT images of each bacterium shows that a single colony (**Figures 5a1-d1**) contains a dense cluster of this cell mass depicted by the high signal intensities throughout the colony. **Figures 5a1-d2** display the 2D images from the planktonic bacteria in pellet form that shows more heterogeneous optical scattering signals from the sediments in the pellets. More dense bacterial deposits are also observed in the pellet, as shown in **Supplementary Video 1**. In **Figures 5a3-d3**, thin bacterial biofilm (~100-200 µm) structures with more heterogeneous scattering intensities and variable thicknesses are observed from the biofilm grown on the substrates. Also, the biofilms of *P. aeruginosa* appeared to form a “mushroom”-like shape, as seen in **Figure 5d3**, and likely contains substantial amounts of water, as indicated by the dark, low-scattering, regions in the OCT images. These water-filled pores have also been reported by other groups as a structural feature of biofilms (Flemming and Wingender, 2010). Better visualization of all three forms can be seen in the **Supplementary 3D volumetric OCT images and videos**.

From these OCT images, quantitative analysis was performed to determine differences in refractive indices and optical attenuation.

#### Refractive index measurements from bacterial species

**Figure 6:**
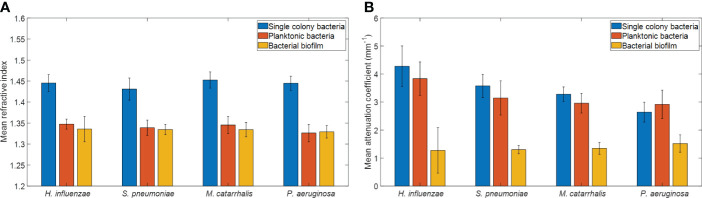
Comparison of **(A)** mean refractive indices and **(B)** mean attenuation coefficients from different bacterial colonies grown on brain heart (BH) agar plates, planktonic form, and biofilm forms. Here, each bar graph represents the mean and the standard deviation from 32 independent measurements.

The refractive index (RI) was extracted by computing the ratio of the optical and geometrical thicknesses of the sample obtained from a cross-sectional OCT image (as described in the methods section) and compared across bacterial species. **Figure 6A** shows the mean RIs of the different bacterial species in the three growth environments. In addition, mean RIs of the planktonic form and biofilms were determined, resulting in values ranging from 1.31 to 1.34. Based on the one-way ANOVA test (*p* < 0.05), the RIs of the colonies had a significantly higher mean RI (~1.44 ± 0.02) than the mean RI of both planktonic form and biofilms (both ~1.33 ± 0.02) across all four of the bacterial species. RI is dependent on the density of cells. Higher RI was observed for the denser single bacterial colonies compared to the more heterogeneous biofilms that contain liquid-filled pores and channels. Bacteria within biofilms are assembled in microcolonies encased by an EPS matrix and separated by water channels, which results in a less dense packing of cells within that matrix and a lower RI. The mean RI from planktonic bacteria and biofilms are almost identical for all bacterial species due to the presence of water in both forms, as shown in **Tables 3**, **4**.

**Table 3:** Overall mean (n = 32 for each independent measurement) and statistical comparison of RI and AC for the different bacterial species in all three growth forms using the one-way ANOVA test.

	Mean ± SD at 1325 nm OCT		*p*-value	
Environment	Refractive index	Attenuation coefficient (mm^-1^)	Refractive index	Attenuation coefficient
Colony			<0.001	<0.001
*H. influenzae*	1.45 ± 0.02	4.25 ± 0.68		
*S. pneumoniae*	1.43 ± 0.03	3.58 ± 0.41		
*M. catarrhalis*	1.45 ± 0.02	3.28 ± 0.25		
*P. aeruginosa*	1.44 ± 0.02	2.91 ± 0.36		
Planktonic			<0.001	<0.001
*H. influenzae*	1.35 ± 0.01	3.82 ± 0.39		
*S. pneumoniae*	1.34 ± 0.02	3.12 ± 0.51		
*M. catarrhalis*	1.35 ± 0.02	2.95 ± 0.26		
*P. aeruginosa*	1.33 ± 0.02	2.64 ± 0.28		
Biofilm			0.56	<0.001
*H. influenzae*	1.34 ± 0.03	1.23 ± 0.17		
*S. pneumoniae*	1.33 ± 0.01	1.29 ± 0.08		
*M. catarrhalis*	1.33 ± 0.02	1.36 ± 0.17		
*P. aeruginosa*	1.33 ± 0.01	1.53 ± 0.16		

Data is statistically significant if *p* < 0.05 and is not significant if *p* ≥ 0.05.

**Table 4:** Inter-group comparison of p-values among bacterial species in colony, planktonic, and biofilm forms by one-way ANOVA with *post-hoc* Tukey’s HSD test.

Intergroup comparisons	*p*-value							
	Otitis media-causing bacterial species							
	*H. influenzae*		*S. pneumoniae*		*M. catarrhalis*		*P. aeruginosa*	
	RI	AC	RI	AC	RI	AC	RI	AC
Colony vs Planktonic	<0.001	<0.001	<0.001	<0.001	<0.001	<0.001	<0.001	<0.001
Colony vs Biofilm	<0.001	<0.001	<0.001	<0.001	<0.001	<0.001	<0.001	<0.001
Planktonic vs Biofilm	0.089	<0.001	0.621	<0.001	0.052	<0.001	0.867	<0.001

Data is statistically significant if *p* < 0.05 and is not significant if *p* ≥ 0.05.

#### Attenuation coefficient measurements from bacterial species

In addition, a depth-resolved pixel-wise single scattering attenuation coefficient algorithm (Vermeer et al., 2014) was applied to the 2D OCT images for each bacterial species in colonies, planktonic form, and biofilm. To determine if OM-causing bacteria scatter light differently based on species and growth conditions, the mean AC of all samples was computed using a custom-developed MATLAB script. **Figure 6B** shows the overall mean ACs and standard deviation of the different bacterial species in the three growth environments.

**Table 3** summarizes the statistical comparison (one-way ANOVA) of the mean RI and mean AC for the different bacterial species in all three growth forms. Comparing the mean values of ACs, the growth of bacteria from colonies to planktonic cells, in liquid cultures, and then to biofilms show substantial differences. In all cases, bacterial colonies grown on agar plates demonstrate a higher AC, while each biofilm result in the lowest AC (**Table 3**). **Tables 4** and **5** show the intra-group comparison and statistical significance (p-value) among bacterial species in colony, planktonic, and biofilm forms and between species in all three forms using the one-way ANOVA with *post-hoc* Tukey’s HSD test, respectively, to distinguish the significant differences between pairs of means. The RI measurements were almost identical between planktonic cells (~1.33-1.35) and biofilms (~1.33-1.34) showing no significant differentiation, and likely due to the presence of water (RI ~1.33) in both forms. However, the AC measurements show significant differences across all growth states for each species (**Table 4**), with each species’ colonies displaying the highest AC values compared to its planktonic cells and biofilms. Moreover, across species (**Tables 3**, **5**), *P. aeruginosa* displays the smallest AC in both colony and planktonic forms. While *H. influenzae* biofilms have significantly smallest AC values (1.23 ± 0.17) across all species, and *P. aeruginosa* biofilms generate the highest AC value (1.53 ± 0.16). Overall, the AC measurements can differentiate all bacterial species in colony form, but can only differentiate *H. influenzae* from the other species within planktonic form, and *P. aeruginosa* biofilms from the other bacterial biofilms. A significant difference is also observed between *H. influenzae* and *M. catarrhalis* biofilms (p < 0.003, **Table 5**). Additionally, all species’ biofilms appear to be heterogeneous. This can be visualized in the AC maps (**Supplementary Figure 3**) and **Supplementary Video 3** as different regions, within the same biofilm, displaying various degree of AC values.

**Table 5:** Intra-group comparison of p-values among colonies, planktonic form, and biofilms by one-way ANOVA with *post-hoc* Tukey’s HSD test.

Intergroup comparisons	*p*-value					
	Between colonies		Between planktonic form		Between biofilms	
	RI	AC	RI	AC	RI	AC
*H. influenzae* vs *S. pneumoniae*	0.029	<0.001	0.226	<0.001	0.996	0.291
*H. influenzae* vs *M. catarrhalis*	0.722	<0.001	0.967	<0.001	0.992	0.003
*H. influenzae* vs *P. aeruginosa*	0.999	<0.001	<0.001	<0.001	0.553	<0.001
*S. pneumoniae* vs *M. catarrhalis*	<0.001	0.045	0.512	0.224	0.999	0.331
*S. pneumoniae* vs *P. aeruginosa*	0.040	<0.001	0.032	0.112	0.695	<0.001
*M. catarrhalis* vs *P. aeruginosa*	0.648	<0.001	<0.001	0.987	0.732	<0.001

Data is statistically significant if *p* < 0.05 and is not significant if *p* ≥ 0.05.

## Discussion

An OM diagnostic tool that can provide additional information about the bacterial otopathogen, including detecting and characterizing the bacterial state (i.e., effusions (planktonic) versus biofilms), would help better inform treatment; including the administration of antibiotics. Herein, the detection capabilities of both RS and OCT were explored independently but under similar experimental conditions to highlight the potential for these two modalities to serve as a future dual-modality tool capable of achieving this diagnostic goal. Both light-based technologies offer different but complementary mechanisms to characterize differences in growth states of OM-causing bacteria and, in most cases, offer a means for differentiation across bacterial species.

First, the differences in the biochemical composition of four OM-causative bacterial species studied herein can be seen using RS. Furthermore, with above 93% sensitivity and specificity, RS can detect feature-specific chemical and structural changes that occur during the growth of the OM-causing bacteria from colonies to planktonic form and finally, biofilms. In particular, *P. aeruginosa* is known to secrete toxins such as pyocyanin as a defense mechanism to safeguard itself within biofilms and promote extracellular DNA (eDNA) release (Das and Manefield, 2012). The Raman signatures of pyocyanin have been well studied by other researchers who noted strong bands at ~1354 and 1610 cm^-1^ (Bodelón et al., 2016; Turasan, 2020). Biofilms of *P. aeruginosa* also display this prominent peak at 1355 cm^-1^, as seen in **Figure 1D**, indicating the secretion of this toxin within three days of the biofilm growth. Also seen, across the three growth forms, is a shift in the Amide I (C=O stretch) Raman band between 1640 – 1680 cm^-1^ (Maquelin et al., 2000; AlMasoud et al., 2021). In particular, the colonies and planktonic form of *P. aeruginosa* Amide I band peak reside at ~1670 cm^-1^. However, for the biofilms, this peak is shifted to ~1659 cm^-1^. The ~1610 cm^-1^ Raman peak of pyocyanin could be contributing to this peak shift associated with the *P. aeruginosa* biofilm formation. Moreover, the chemical secretion, and its corresponding Raman peak at 1355 cm^-1^, provides a means to distinguish *P. aeruginosa* biofilms from its colony and planktonic form. This key Raman peak also allows for differentiation from the other OM-causing bacterial species studied herein, as seen in **Figure 2**, with 100% specificity and sensitivity.

For *S. pneumoniae*, the key differences between the biofilm and planktonic forms are seen in **Figure 1B** as changes in the protein/lipid peak ratio at 1095 cm^-1^ (C-N vibrational bonds) and the skeletal acyl backbone of lipid Raman peak at ~1124 cm^-1^ (C-C: transconformation) (He et al., 2014). The planktonic form exhibits a stronger Raman intensity at 1095 cm^-1^ compared to the biofilms. Also, a conformational change of the protein/lipid is observed, where the colonies exist in a -’*cis’* configuration (1660 cm^-1^) while the planktonic and biofilms exist in the *‘trans’* (1670 cm^-1^) configuration (Talari et al., 2015). These changes suggest that one potential mechanism in the formation of *S. pneumoniae* biofilms is associated with membrane lipid structural changes as the bacteria adapts to biofilm growth. Furthermore, the relative redistribution in the intensity of the nucleic acid content (~740–832 cm^-1^) of the *S. pneumoniae* biofilms provides a discriminatory factor for this bacterium from the other three OM-causing bacteria. This redistribution is seen in **Figure 2** as a higher relative Raman intensity between 800-830 cm^-1^, and potentially relates to higher levels of extracellular DNA (eDNA) release compared to the other bacteria. The release of eDNA is important for the initial adhesion and aggregation of bacterial cells toward biofilm formation. This biomolecule is reported to be released spontaneously during the formation of *S. pneumoniae* biofilms (Moscoso et al., 2006). Also, *S. pneumoniae is* a gram-positive bacterium and the only one of the four studied herein. Therefore, differences are seen in the Raman bands of its agar colonies between 710- 730 cm^-1^ could relate to its cell wall components (**Figure 4**). Unlike the other species whose nearest Raman peak resides at ~725 cm^-1^ and relates to DNA content (Guicheteau et al., 2006), the Raman spectra of this bacterium contain a distinct Raman peak at 716 cm^-1^ suggesting the presence of phospholipid-containing choline biochemical signatures (Krafft et al., 2005). Researchers have reported that this bacterium cell wall includes lipoteichoic acid-choline moieties (Meiers et al., 2014). Therefore, the distinct peak at 716 cm^-1^, suggests the Raman spectral feature of the choline contained within its cell walls, which is not present in the cell walls of the other bacteria. In addition to the 716 cm^-1^ spectral feature, other differences in the phospholipid signatures can be seen in the RS regions of 1094-1126 cm^-1^ and 1294-1304 cm^-1^ in **Figure 4**. These differences are potentially related to the bio-composition of the outer membrane of gram-positive versus gram-negative bacteria. Altogether, these three spectral differences allow for the differentiation of *S. pneumoniae* colonies, grown on BHI agar, from the other three bacterial species.

Similar to *S. pneumoniae*, *M. catarrhalis* biofilms also demonstrates lipid structural changes as shown in the reduction in relative Raman intensity at 1098 cm^-1^, but maintains relatively similar Raman intensities at 1124 cm^-1^ compared to its planktonic form (**Figure 1C**). Also, in differentiating the agar colonies from the other environments, the Amide I Raman peak resides at 1660 cm^-1^ for the colonies compared to 1670 cm^-1^ for the planktonic form and biofilms. However, the main distinguishing feature of this bacterium to differentiate the planktonic form and agar colonies from its biofilms relates to the Raman peak at ~1554 cm^-1^. This peak potentially relates to elevated levels of tryptophan release during DNase production (Hussein, 2016) and can be used to differentiate *M. catarrhalis* biofilms from the other OM-causing bacterial species (**Figure 2**).

Raman spectroscopy is also able to differentiate all three growth forms of *H. influenzae*. The differences can be seen in **Figure 1A** in the Raman regions between 1643-1650 cm^-1^, 1342-1373 cm^-1^, and ~666 cm^-1^. The biofilms of *H. influenzae* differ from its planktonic form in the Raman spectral regions of 666 cm^-1^ and 1342-1373 cm^-1^ relating to the redistribution of amino acid and proteins in the 1500-1700 cm^-1^ region (Guicheteau et al., 2006). One potential cause of these redistributions may be the regulation of proteins in the formation of biofilms. A proteomic study conducted by Post *et al.* reported that there is a downregulation of proteins associated with energy metabolism and protein synthesis, but those involved in fatty acids, DNA metabolism, and transcription are upregulated when *H. influenzae* exists in biofilm form (Post et al., 2014). These protein biochemical changes can be detected *via* RS and allow for the differentiation of *H. influenzae* from the other bacterial species (**Figure 2**).

Altogether, RS can characterize the biochemical differences that occur between growth forms of the same species as well as the differences in bio-compositions across species. Across species, RS can differentiate the four causative species from each with other with specificities greater than 96% in all three growth forms. This is important in the case of OM detection where the different causative species and growth states (i.e., planktonic form within effusion versus biofilms) may cause varying response to antibiotics. Thus, knowing more information about the disease can better inform treatment. However, the biochemical “fingerprint” is only one aspect of bacterial characterization. Its morphological structure, as it inhabits an environment, can also be used for characterization and differentiation. In particular, biofilms are known to be inherently heterogeneous (Hall-Stoodley et al., 2008; Hall-Stoodley and Stoodley, 2009). Therefore, OCT, an imaging modality, has the added benefit of visualizing and spatially assessing the morphological changes of the bacteria in its colony, planktonic form, and biofilm forms, with fast acquisition times, to better inform species and growth identification.

OCT is a non-invasive and non-destructive label-free technique that requires no sample preparation. Herein, it was utilized to assess whether morphological differences can be detected *via* the RIs and/or ACs for each of the four OM-causing bacterial species. As the OM-causing bacterial species are grown from colonies to biofilms, there are observed differences in the light scattering optical properties based on varying morphological differences. It is expected, that samples with thicker layers or membranes, denser or clustered cells, or ones which contains higher amounts of biochemical secretions will result in larger light scattering events. In general, single colony bacteria are highly dense clusters of cells, and factors such as how tightly packed these colonies are as well as what biochemical they secrete can impact its optical properties. Planktonic cells are free-floating cells in a liquid matrix, and are responsible for initiating biofilm formation in response to a nutrient-rich environment by undergoing various metabolic response pathways, also known as quorum sensing (O’Toole et al., 2000) which can impact its optical properties based on the secretory biochemicals and the density of cells. Whereas, biofilms are dynamic but represent a stable point in the bacterial lifecycle and contain distributed structures with water pores and channels; and thus, contributing to a much lower density (Wang et al., 2011) across the extracellular polymeric substance (EPS) matrix. Therefore, biofilms are expected to produce less scattering than the denser planktonic form or colonies (Hou et al., 2019); due to the presence of a mixed environment of bacterial aggregates and an EPS matrix with high water content (also known as a highly hydrated gel) (Flemming and Wingender, 2010) in the biofilm. Within this study, these structural differences were measured *via* RI and AC, where RI and AC provide information about how light is transmitted and scattered, respectively, through a sample. Herein, a decrease in RI of the bacterial colonies compared to its biofilms’ counterparts is observed. Moreover, the measured RI for each species’ biofilms ranged from ~1.33-1.34 which is similar to the RI of water (~1.33), which further confirms that the biofilm matrix contains high water content. Also, the measured RIs of the biofilms in this study, particularly for *P. aeruginosa*, is comparable to previous work shown by Bakke et al. who reported a mean RI of ~1.35 for *P. aeruginosa* biofilms (Bakke et al., 2001). The measured RIs for the colonies also correspond well with the reference RI values of bacterial colonies in a prior study (Balaev et al., 2003). However, the presence of water in the planktonic and biofilm stages minimized the capabilities of RI to differentiate across all growth environments and species. Thus, this study show that the optical scattering differences quantified by the AC measurements can more easily distinguish across growth environments and species in comparison to the RI measurements as shown in **Figure 6B**.

In general, the biofilms exhibit lower AC values compared to the planktonic or colony forms, due to the lower cell density as can be seen in **Figure 5**. *P. aeruginosa* displays the lowest AC in both colony and planktonic forms and *H. influenzae* has highest AC values across all species in both forms. As seen in the **Figure 5**, compared to all other species’ colonies, *H. influenzae* produce smaller and more compact colonies which result in more light scattering events than *P. aeruginosa* bacterial colonies. On the other hand, the 3-days old *H. influenzae* biofilms show the smallest AC across all species, with the 3-days old *P. aeruginosa* biofilms generating the highest AC. All biofilms were grown in the nutrient-rich BHI medium that contained proteose peptone and infusions from the calf brain and beef heart which provided carbon, nitrogen, essential growth factors, amino acids, and vitamins for biofilm formation. *P. aeruginosa* is reported to be one of the greatest biofilm producers (Pericolini et al., 2018) and produces more secretions of a highly diffusible nitrogen-containing compound, known as pyocyanin toxin metabolites (Das et al., 2016), at an early stage (~ within 14 h) of biofilm formation. It has also been reported that pyocyanin promotes eDNA release (Das and Manefield, 2012) in *P. aeruginosa* biofilms by inducing cell-lysis to bacteria cells mediated by the production of hydrogen peroxide (Allesen-Holm et al., 2006), which enhances the *P. aeruginosa* biofilm formation. Therefore, the increase in the mean AC for the *P. aeruginosa* biofilm suggests the presence of these secreted molecules, also confirmed by the RS data, as well as the formation of dense gels of the EPS biofilm matrix, which resulted in more scattering events. Another possible explanation for the dense EPS matrix of *P. aeruginosa* biofilm can be the increase of cellular cyclic-di-GMP (Guttenplan and Kearns, 2013) levels. This increase causes the reduction of the flagella-assisted swarming motility (Shrout et al., 2006; Guttenplan and Kearns, 2013) during the early stages of biofilm formation by transitioning motile *P. aeruginosa* to non-motile bacteria in the biofilm state.

The 2D and 3D images of biofilms from each species also provide more information about the change in signal intensities in relation to the spatial distribution of the matrix. The 3D OCT images are constructed by combining multiple cross-sectional 2D OCT images; thus, these images provide better visualization of the complex internal heterogeneous structures such as bacterial cell aggregates, pores, and water channels inside the biofilm, as shown in **Supplementary Figure 2**. In particular, the obtained images show that the biofilms contain mostly water-filled pores. The pores are noted as relatively dark (low-scattering) regions in the OCT images. It has been theorized, by other research groups, that these channels and pores supply localized nutrients to the growing biofilm (Flemming and Wingender, 2010). Another advantage of OCT imaging is that the AC coefficient maps can characterize the rate of change of OCT signals as a function of depth, which is important given that biofilms have a heterogeneous structure and often grow to thicknesses of hundreds of microns or more. These thicknesses are well beyond the imaging depth of fluorescence and confocal microscopes. The 2D images also show differences in the heterogeneity of the biofilms across species. In particular, *S. pneumoniae* biofilms show less heterogeneity than the other species, while both *M. catarrhalis* and *H. influenzae* show layered biofilms in the bottom layer. On the other hand, the top surface of *P. aeruginosa* is very distinct from the other species and show pores with water channels and morphological differences. Currently, we are performing texture analysis to identify these variations of OCT images among three growth forms and across species.

In summary, OCT provides visualization of the bacterial species in each environment *via* 2D and 3D reconstruction. This visualization and complementary optical properties values provide a quantitative metrics for differentiating across growth environment and species. In particular, the mean attenuation coefficients of bacterial colonies, planktonic form, and biofilms allowed for significant differentiation of each species in the three different growth environments and in most cases across species. Overall, a major advantage of OCT is the depth imaging of each species which allow for better understanding of the bacteria grows within its growth environment.

This study has additional considerations that are worth mentioning. First, in terms of sample preparation and growth methodology, it should be noted that bacterial growth largely depends on the growth media and environments such as CO_2_, temperature, pH, humidity, and substrates. In literature, various culture media, agars, and environmental conditions have been used to grow these bacteria. The experiments in this study followed the standard protocols as mentioned in the Methods section for the optimal growth of bacteria in colonies, planktonic, and biofilm forms. Briefly, nutrient-rich BHI broth and agar were used as culture media for all bacterial species except for *H. influenzae*. For *H. influenzae*, BHI media/agar supplemented with hemin and NAD was used. All the samples were incubated at 37 °C, 5% CO_2_. Also, the initial attachment of planktonic bacteria on the substrate surface is the primary step for growing a bacterial biofilm. Herein, biofilms were grown on calcium fluoride slides (for RS measurements) and poly-d-lysine coated culture dishes (for OCT measurements) for initial adhesion of bacteria and growth of the biofilms. Calcium fluoride was necessary to minimize substrate background noise in the Raman spectra. We know that other growth media and conditions could potentially be used to culture the different species in the various growth forms, which could impact both the biochemical and scattering properties of the cells. Therefore, the media and growth conditions were standardized in this study to minimize the influence of these external factors. Also, planktonic bacteria in BHI media were cultured from the overnight growth of bacteria colonies on BHI agar. The RS and OCT measurements were acquired on dense spun-down pellets obtained from these planktonic bacteria to improve the signal-to-noise of the spectroscopic measurement.

**Figure 7:**
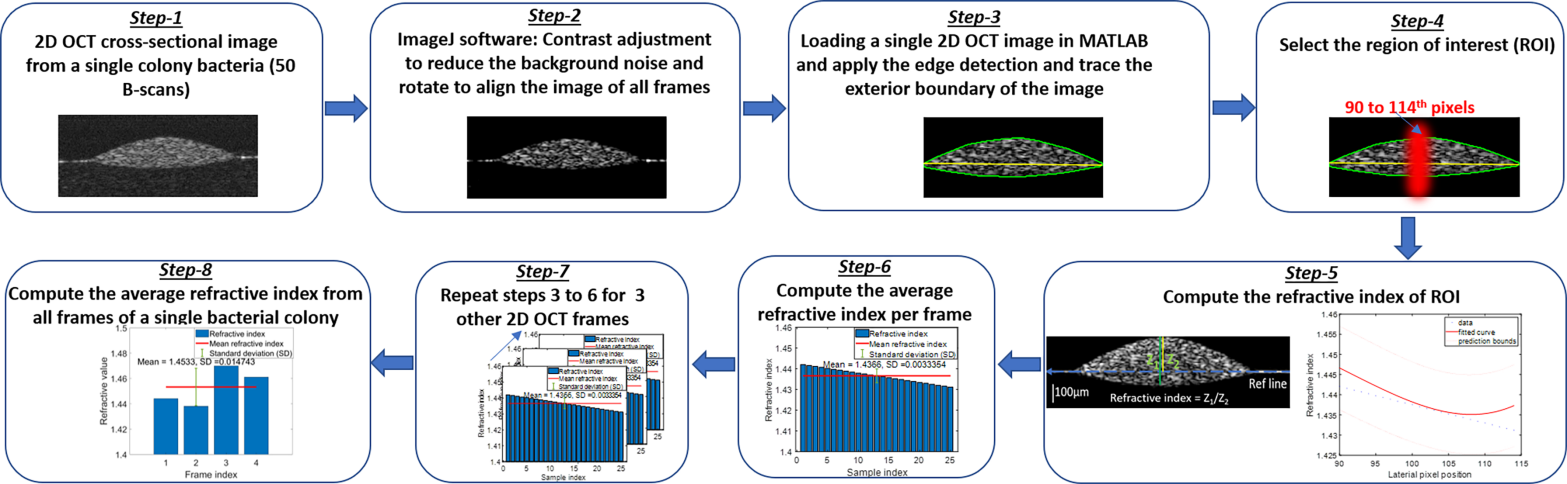
Flow diagram of the refractive index calculation algorithm.

Other factors to consider relate to the methodology used to quantify the RI and AC values, as several methods can be used to calculate these values. Herein, the RI measurement was based on the ratio of the optical thickness and the geometric thickness of the sample from a single OCT B-scan frame as shown in the flow diagram of **Figure 7** (Step - 5). The bulk RI of the sample can be effectively measured with our current SDOCT setup, and the sensitivity of RI is ~0.01. For the AC measurements, a depth-resolved single-scattering algorithm was implemented to determine the intensity variations of adjacent voxels and to recover the amount of attenuation that occurs pixel-wise. The algorithm assumes the complete attenuation of light through the sample and the linear relationship between backscattering and attenuation, which overestimates the AC value at the greater imaging depths of the OCT B-scan images. The thicknesses of our samples were comparatively small (~200 µm), allowing for the overestimation error at greater depths to be negligible, but for thick samples, these errors were mitigated by fitting an exponential curve on averaging the last few pixels of the bottom of the sample (OCT B-scan), also known as the optimized depth-resolved estimation (ODRE) algorithm (Liu et al., 2019).

Overall, these results demonstrate the advantages provided by both Raman spectroscopy and OCT to detect and characterize differences in the biochemical composition and scattering properties of the four primary OM-causing bacterial species. Moreover, the ability to differentiate these species in both their planktonic and biofilm forms provide a valuable dual contrast approach, based on the biochemical and morphological differences, in assessing the presence and growth environment of each bacterial species. With the current clinical standards requiring complex secondary tools such as PCR and nucleic acid sequencing for the identification of bacterial species, and the lack of currently-used tools capable of detecting effusions and biofilms, in real-time, a dual RS-OCT approach has the potential to be a useful multi-modal tool for future *in vivo* detection and assessment of OM. However, translating such technologies for OM diagnosis *in vivo* requires resolving some physiological constraints and design challenges. For instance, acquiring Raman spectra of effusions or biofilms from directly behind the delicate TM with a fiber optic RS probe is challenging. Therefore, we recently developed a prototype of a multimodal RS-OCT system and successfully tested it in *ex vivo* ear models (i.e., clinical effusion under chinchilla TM). This provided an understanding of the specific physiological limitations (improved positioning feedback *via* OCT for RS measurements) and design challenges (i.e. maximum permissible exposure and optical safety limits) for signal collection within the middle ear (Monroy et al., 2022). Currently, we are designing and implementing our 2^nd^ generation RS-OCT for the clinical studies of *in vivo* effusions and biofilms. With additional study and refinement, the diagnostic capabilities of this combined RS-OCT platform may positively impact *in vivo* detection and assessment of OM in the future.

## Methods

### Materials

Nontypeable *Haemophilus influenzae* (Gram-negative, ATCC 49766), *Moraxella catarrhalis* (Gram-negative, ATCC 49143), *Streptococcus pneumoniae* (Gram-positive, ATCC 6301), and *Pseudomonas aeruginosa* (Gram-negative, ATCC 14203) were all purchased from American Type Culture Collection (ATCC) and stored in glycerol at –80°C. Brain heart infusion (BHI) media was purchased from Fisher Scientific and agar, hemin from bovine ≥ 90%, β-Nicotinamide adenine dinucleotide hydrate (NAD), and paraformaldehyde (PFA) were purchased from Sigma Aldrich. The Filmtracer™ LIVE/DEAD™ Biofilm Viability Kit was obtained from ThermoFisher Scientific. Phosphate buffer saline (1x) was prepared in distilled water and autoclaved.

#### Bacteria colonies cultured on BHI agar

Propagation methods as recommended by ATCC were used for each planktonic bacterial species in preparing the cultures, which involved streaking of each culture on separate agar plates and incubating those plates at 37°C under 5% CO_2_. BHI media was used as culturing media for *M. catarrhalis*, *S. pneumoniae* and *P. aeruginosa*. BHI is a nutrient-rich media, and hence, can be used to culture a variety of fastidious microbes. To prepare BHI agar and media solution, 9.25 g BHI media powder was added to separate bottles containing 250 mL distilled (DI) water. In one bottle, 3.75 g of agar was added. Both bottles were autoclaved (250°F) for 30 min. The agar plates were then prepared in a biosafety hood by pouring the BHI media/agar solution into separate Petri dishes. To culture *H. influenzae*, the agar solution was supplemented with hemin (1 mg/mL) and NAD (10 mg/mL). For *H. influenzae* biofilm culture, *H. influenzae* test media (HTM, ThermoScientific Remel) broth supplemented with hemin and NAD was used. The plates were allowed to set overnight and were checked for visual contamination prior to use. Each bacterium was removed from the –80°C freezer and immediately streaked on separate BHI agar plates, in triplicates, with a sterile stick. The streaked plates were incubated overnight at 37°C under 5% CO_2_ prior to characterization *via* both RS and OCT methodologies described below.

#### Liquid cultures in BHI media

Liquid cultures (n = 3 for RS and n = 6 for OCT) for each bacterium were prepared following the overnight growth of bacteria colonies on BHI agar. An inoculation loop was used to remove a single colony from the agar plate, which was then placed into a centrifuge tube with 10 mL BHI media (with NAD + hemin supplemented for *H. influenzae*). The liquid cultures were then incubated for 24 h at 37°C under 5% CO_2_. Following incubation, 1 mL of solution was extracted and centrifuged for 5 min at 3.3k rcf. The supernatant was removed and re-suspended in an equal volume of 1x PBS. This washing method was performed twice before the pellet was removed and characterized *via* RS and OCT.

#### Biofilm growth over 3 days

Biofilms (n = 3 per bacterium) were then prepared from 1 mL of the liquid cultures prepared above, diluted in fresh BHI media (5 mL) at a 1:6 volumetric ratio. This solution was then incubated (37°C, 5% CO_2_) for 3 h in order to reach the mid-log phase. This mid-log growth suspension was then further diluted at 1:2500 volumetric ratio with new BHI media. To characterize the biofilms *via* RS, 2 mL of this diluted solution was transferred to a glass-bottom Petri dish containing a calcium fluoride (CaF_2_) Raman substrate (Crystran Ltd, United Kingdom). For OCT characterization, 200 µl of the diluted solution was transferred into a poly-d-lysine coated microscope slide (Azer Scientific), and the slide was kept inside a Petri dish. The Petri dishes were then incubated (37°C, 5% CO_2_) for three days with new BHI media exchanged once per day using a pipette to aspirate the media from the corner of the dish without disrupting the biofilm, and to maintain the bacterial viability in the biofilm. After three days, the media was aspirated, and the calcium fluoride-containing Petri dishes were washed three times with 1x PBS and fixed with 4% PFA followed by a final wash with DI water prior to RS measurements. For the OCT measurements, the excessive media from the biofilms grown on the poly-d-lysine slide surfaces were aspirated, and the slides were washed with DI water. A LIVE/DEAD staining was conducted on separate biofilms to determine bacterial viability within the biofilm (**Supplementary Figure 4**).

#### Raman spectroscopy characterization

All RS measurements were conducted on a benchtop inVia™ confocal Raman microscope (Renishaw, United Kingdom). Agar culture colony measurements were taken under 100x objective (NA 0.85) with 24 mW power and an integration time of ~15 sec. For each agar plate, 10 measurements were taken per colony and three colonies per plate. Raman spectra were also taken of the agar plates alone, and it was determined that both agars did not influence the Raman spectra of the bacterial colonies (**Supplementary Figure 5**). The cell pellets separated from each liquid culture were placed in a stainless steel well to avoid interference from substrate-related Raman bands. Three pellets from three separate cultures were obtained. Raman measurements were then taken under a 50x objective (NA 0.75) with ~ 25-30 mW power for 15 sec. Three biofilms per bacterial species were grown from three separate cultures and measured on CaF_2_ slides. Raman measurements were acquired from 10 different locations across the biofilm under a 100x objective for 45 sec with ~25 mW power at the objective. All acquired Raman spectra were pre-processed for intensity calibration, fluorescence subtraction, noise-smoothing, and mean normalization in the MATLAB coding environment using protocols previously developed (Lieber and Mahadevan-Jansen, 2003). This included noise filtering using a Savitzky-Golay smoothing filter and fluorescence subtraction using a modified polynomial fitting approach before normalization using standard normal variate. SMLR was then used to identify key differences within the same species across growth environments (i.e., agar, liquid, biofilms) as well as across species comparisons of agar colonies, planktonic form (i.e., pelleted cells from liquid cultures), and biofilms. A sparsity of 1 and k-fold cross-validation of 5 was used to determine the wavenumber variables that contributed more to the sample classification based on their average weights. Also, where not specifically stated, the Raman peaks were interpreted using the well-cited Raman spectroscopy of biological tissue library provided by Talari *et al.* (Talari et al., 2015).

#### Optical coherence tomography characterization

A custom-built 1325 nm benchtop spectral-domain OCT system (**Supplementary Figure 1**) was utilized to capture cross-sectional and 3D images of single colony bacteria on agar, pelleted planktonic bacteria in liquid culture, and biofilm. In brief, the benchtop OCT system utilized a broadband superluminescent diode (Thorlabs, New Jersey) source with a center wavelength of 1325 nm and a bandwidth of 100 nm, which provided an axial resolution of ~8 µm (air). The lateral resolution was ~16 µm in air. A 1024-pixel InGaAs line-scan camera (Goodrich, North Carolina) was used in the spectrometer, delivering an optical imaging depth of ~2.2 mm. The optical power applied to the sample was less than 10 mW. A total of 50 B-scans per volume were acquired from five single colonies of bacteria on each culture plate. Similar OCT measurements were performed on the agar plates alone to validate differences in the optical properties between the colonies and the agar plate (**Supplementary Information**). Six bacterial pellets were removed from each liquid cultured bacteria and were placed on a microscope slide. From the six pellets, 10 B-scans per volume were acquired per bacterium. To characterize the biofilms, 1000 A-scans per B-mode image and 100 sequential B-mode frames were captured from each biofilm, at three locations, using a pair of galvanometer scanners (Thorlabs, New Jersey).

##### Refractive index computation algorithm

The RI of biological cells and microorganisms is important for characterizing the interactions between light and biological scatterers in samples. Differences in RI have been used to detect variations of chemical content inside cells and their distribution (Wang et al., 2011), differentiate growth rates of living cells (Zibaii et al., 2010), and distinguish between normal and malignant tissues (Grigorev et al., 2020) by measuring and comparing their RI values. In OCT, a single depth scan (A-scan) represents the depth-dependent intensity profile of the sample along the beam path. The optical thickness of this sample in an A-scan is the product of the group RI (n_g_) and the geometrical thickness of the sample. The n_g_ depends on the wavelength dependence of the phase RI, n_p_, as: ${n_{g}=\;n_{p}-\lambda_{0}\;\frac{d}{d\lambda }\left(n_{p}\right)|}_{\lambda_{0}}$ , where λ_0_ indicates the central wavelength of the light source in the OCT system. Thus, in practice, n_g_ ≈ n_p,_ as the derivative term is small and negligible (Tearney et al., 1995). Therefore, we can directly compute the RI by the optical path-length matching method (Tearney et al., 1995), where RI can be extracted by computing the ratio of the optical and geometrical thicknesses of the sample obtained from a cross-sectional OCT image.

To analyze the variations of refractive indices among all bacterial species in a colony, planktonic (in pellet form), and biofilm forms, ImageJ and a MATLAB-based program were used, developed, and applied to compute the average RI from all samples. **Figure 7** shows the graphical representation of the flow diagram for the RI computation by optical path-length measurement using OCT on a single bacterial colony (Tearney et al., 1995). The 2D reconstruction of each OCT data set consisted of 1024 × 512 (rows and columns) pixels, processed by MATLAB and saved in *tiff* format. ImageJ software was used to reduce background noise from the processed 2D OCT images and to generate 3D OCT images. The MATLAB algorithm binarized each input image (2D OCT) employing Canny’s edge detection to look for the local maxima of the gradient of the input image and to trace the exterior boundary of the image. Then, a window size of 25 pixels in the lateral direction was selected around the peak intensity position of each 2D OCT image, followed by the computation of RI by taking the ratio of the optical thickness (z_1_) and the geometrical thickness (z_2_) for each individual bacterial colony from a single OCT B-scan frame as illustrated in **Figure 7**. Next, the average RI per frame was computed, and the same procedure was repeated for all four frames and averaged to obtain the mean refractive index from a single bacterial colony for the desired window size of the 2D OCT image. The same procedure was repeated for multiple single colonies of bacteria on each culture plate, all bacterial pellets, and biofilms to compute the mean refractive index of bacteria species in all three forms.

##### Attenuation coefficient computation algorithm

Additionally, a depth-resolved pixel-wise single scattering attenuation coefficient algorithm (Vermeer et al., 2014) was applied to each 2D OCT image of bacteria in colony, planktonic, and biofilm forms to compute the mean attenuation coefficient of each image. Attenuation coefficients (AC) indicate the rate of optical signal decay due to absorption and scattering of light by a sample. However, for biological applications, OCT systems typically uses near-infrared wavelengths, and hence, the optical attenuation response of the biological sample is governed largely by scattering rather than absorption.

**Figure 8:**
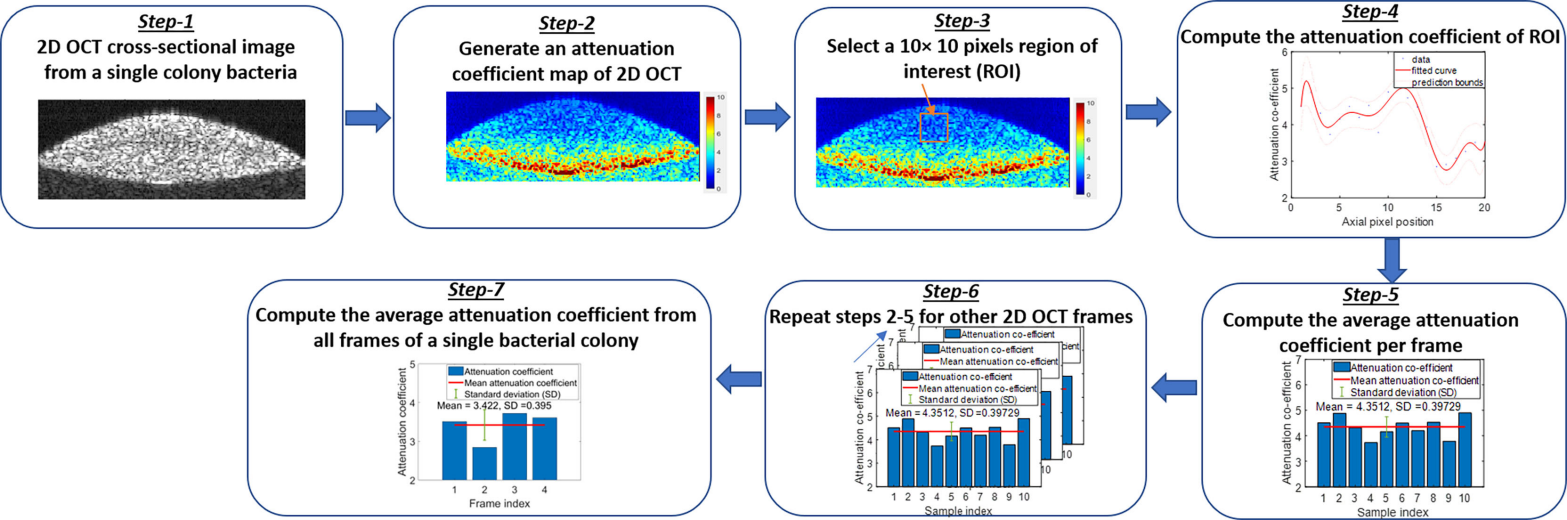
Flow diagram of the attenuation coefficient calculation algorithm.

The single scattering model assumes only a single occurrence of backscattering of photons in weakly scattering samples or superficial layers of a high scattering samples. **Figure 8** shows the graphical representation of the flow diagram for the AC algorithm on a single bacterial colony. A MATLAB-based program was used to calculate the average AC from all samples. Attenuation coefficients indicate the rate of optical signal decay due to absorption and scattering of light from the sample. ACs facilitated differentiation among colony, planktonic, and biofilm forms and across most species due to their different optical scattering properties. The attenuation coefficient *µ(x)* is calculated based on two assumptions: (1) all the light is extinguished within the OCT image depth range, and (2) the backscattered light is a fixed portion of the attenuation coefficient. Assuming a constant intensity over a pixel, the attenuation coefficient *µ(x)* was estimated from the OCT intensity images (*I(x)*), divided by the total number of pixels along an A-scan of each 2D OCT image, n = 80-120, and the pixel size Δ in millimeters by:


(1) $$\mu \left(x\right)=\;\frac{I\left(x\right)}{2\Delta \sum_{x+1}^{n}I\left(x\right)}.$$

The MATLAB algorithm generated an AC map for the entire OCT image using equation (1). Then a window size of 10 × 10 pixels was selected to compute the average AC per frame. Next, the same procedure was repeated to all frames and averaged to obtain the mean AC from a single bacterial colony for the desired window size of the 2D OCT image. The same procedure was repeated for multiple single colonies of bacteria on each culture plate, as well as all bacterial pellets and biofilms to compute the mean AC of bacteria species in all three forms.

##### Statistical analysis of OCT images

The obtained RI and AC results were statistically analyzed by one-way ANOVA and intergroup comparisons. All the statistical analysis was performed using Minitab statistical software (version 19) and MATLAB. Differences were considered as statistically significant at *p*-value < 0.05. The normality of the data was checked by the Anderson–Darlington test (Öner and Deveci Kocakoç, 2017; Stephens, 2017) (**Table S1**). Finally, a comparison of the mean values was performed using one-way ANOVA with a *post-hoc* Tukey’s Honestly Significance Difference (HSD) test.

## Data availability statement

The original contributions presented in the study are included in the article/**Supplementary Material**. Further inquiries can be directed to the corresponding author.

## Author contributions

AKL and FRZ were involved in designing, performing, and analyzing the experiments and results, and drafting the manuscript. KS was involved in performing the experiments and analyzing the results. SF was involved in drafting and editing the manuscript. GLM, HC, and JW were involved in editing the manuscript. AM-J. and SAB were involved in designing the experiments, editing the manuscript, and acquiring funding. AKL and FRZ are co-first authors. All authors contributed to the article and approved the submitted version.

## Funding

The authors appreciate the support from the National Institutes of Health (NIBIB: R01EB028615).

## Acknowledgments

The authors would like to acknowledge support from the Imaging Technology Group and Microscopy Suite of the Beckman Institute for Advanced Science and Technology for generating CLSM images from their confocal laser scanning microscope (CLSM) system (Leica TCS SP8 CLSM, Leica Microsystems Heidelberg GmbH, Manheim, Germany).

## Conflict of interest

SAB, GLM and JW have disclosed and patented intellectual property with the University of Illinois Urbana-Champaign related to the optical detection and characterization of OM. AM-J has disclosed and patented intellectual property with Vanderbilt University related to the optical detection and characterization of bacteria. SAB is a co-founder and Chief Medical Officer of PhotoniCare, Inc., which is developing OCT for the detection and diagnosis of OM.

The remaining authors declare that the research was conducted in the absence of any commercial or financial relationships that could be construed as a potential conflict of interest.

## Publisher’s note

All claims expressed in this article are solely those of the authors and do not necessarily represent those of their affiliated organizations, or those of the publisher, the editors and the reviewers. Any product that may be evaluated in this article, or claim that may be made by its manufacturer, is not guaranteed or endorsed by the publisher.
