# Supplementary material for: Differentiation of otitis media-causing bacteria and biofilms via Raman spectroscopy and optical coherence tomography
Source: Front Cell Infect Microbiol. 2022 Aug 10;12:869761. doi: 10.3389/fcimb.2022.869761 (PMC9400059; doi:10.3389/fcimb.2022.869761)
Supplement: Supplementary file 1 [file DataSheet_1.docx]

Supplementary Material

# Supplementary Data

## Optical Coherence Tomography workflow and characterization

With a custom-built benchtop OCT system operating at a center wavelength of 1325 nm as shown in **Supplementary Figure 1a**, all four otopathogenic bacteria were characterized and analyzed in colony, planktonic, and biofilm forms. **Supplementary Figure 1b** represents the workflow for the OCT imaging system. Briefly, the cross-sectional OCT images were obtained from the single colony bacteria, bacterial pellet and biofilm using the spectral domain OCT system. From these 2D OCT images, 3D volumetric OCT images (**Supplementary Figure 2**) and videos were generated. We used pseudo-color for the videos to get better visualization of a bacterial colony, pellet, and biofilm. The single colony bacteria video shows the denser cluster of bacteria, whereas the planktonic bacteria or free-floating bacterial cells show bacterial deposit on the substrate after spinning it down and the empty spaces indicate the presence of water in OCT. Also, a portion of the biofilm was presented in 3D video where we can see the structure of EPS matrix in light yellow, whereas the space inside the EPS indicates the presence of pores and water in OCT, the bright yellow surface is the substrate. Biofilms contain distributed structures with pores and water channels contributing to a much lower density (Wang et al. 2011) across the extracellular polymeric substance (EPS) matrix. The water-filled EPS matrix present in the biofilm produces less scattering than the denser planktonic form or colonies.

Moreover, quantitative analysis was performed on the 2D cross-sectional OCT images to analyze differences in refractive indices and optical attenuation.

## Attenuation coefficient map of biofilm

The attenuation coefficient (AC) map characterizes the rate of change of OCT signals as a function of depth. The heterogeneous structure of biofilms, due to the presence of EPS components and bacteria throughout the biofilm, can be visualized as different AC values in an AC map (represented with the Jet colormap in MATLAB) as shown in **Supplementary Figure 3**. Pores are more visible for the *M. catarrhalis* biofilm as shown in **Supplementary Figure 3d**.

## Anderson-Darling normality test

The normality of the RI and AC data was checked by the Anderson–Darlington test. The Anderson–Darling test is a statistical normality test that compares the empirical cumulative distribution function (ECDF) of a given sample data (n) with the distribution expected if the data are normal. If the observed difference is sufficiently large, the null hypothesis of normal distribution of data will be rejected. A small Anderson-Darling value indicates that the distribution fits the data better.

The Anderson-Darling normality test is defined as:

Null hypothesis, H_0_: The data follow a normal distribution if p-value > 0.05.

Alternate hypothesis, H_1_: The data do not follow a normal distribution if p-value < 0.05.

The Anderson–Darling (AD) statistics A^2^ is derived from the following equation:

$\boldsymbol{A}^{\boldsymbol{2}}\boldsymbol{= -n-}\frac{\boldsymbol{1}}{\boldsymbol{n}} \sum_{\boldsymbol{i=1}}^{\boldsymbol{n}} \left( \boldsymbol{2}\boldsymbol{i-1} \right)\left[ \mathbf{ln(} \boldsymbol{f}\left( \boldsymbol{y}_{\boldsymbol{i}} \right)\boldsymbol{)+}\ln\left( \boldsymbol{1-f}\left( \boldsymbol{y}_{\boldsymbol{n+1-i}} \right) \right) \right]$ (1)

where $f\left( y_{i} \right)=\emptyset\left( \frac{y_{i}- \bar{x}}{\sigma} \right)$, which is the cumulative probability of the standard normal distribution and y_i_ denotes the sorted data in the ascending order.

Next, the adjusted A^2^ test statistic is calculated to obtain critical values for sample size of n = 32:

$\boldsymbol{A}_{\boldsymbol{adj}}^{\boldsymbol{2}}\boldsymbol{=}\boldsymbol{A}^{\boldsymbol{2}}\left( \boldsymbol{1+}\frac{\boldsymbol{0.75}}{\boldsymbol{n}}\boldsymbol{+}\frac{\boldsymbol{2.25}}{\boldsymbol{n}^{\boldsymbol{2}}} \right)$ **. (2)**

Then the calculated p-value for Anderson-Darling test is:

***p* =** $\left\{ \begin{aligned} \begin{aligned} \boldsymbol{1-}\exp\left( \boldsymbol{-13.436+101.14}\boldsymbol{A}_{\boldsymbol{adj}}^{\boldsymbol{2}}\boldsymbol{-223.73}{\boldsymbol{(A}_{\boldsymbol{adj}}^{\boldsymbol{2}}\boldsymbol{)}}^{\boldsymbol{2}} \right)\boldsymbol{& if}\boldsymbol{A}_{\boldsymbol{adj}}^{\boldsymbol{2}}\boldsymbol{\leq0.2} \\ \boldsymbol{1-}\mathbf{exp}\boldsymbol{(-8.318+42.796}\boldsymbol{A}_{\boldsymbol{adj}}^{\boldsymbol{2}}\boldsymbol{-59.938(}{\boldsymbol{A}_{\boldsymbol{adj}}^{\boldsymbol{2}}\boldsymbol{)}}^{\boldsymbol{2}}\boldsymbol{) if 0.2 <}\boldsymbol{A}_{\boldsymbol{adj}}^{\boldsymbol{2}}\boldsymbol{\leq0.34} \\ \exp\left( \boldsymbol{0.9177-4.279}\boldsymbol{A}_{\boldsymbol{adj}}^{\boldsymbol{2}}\boldsymbol{-1.38}{\boldsymbol{(A}_{\boldsymbol{adj}}^{\boldsymbol{2}}\boldsymbol{)}}^{\boldsymbol{2}} \right)\boldsymbol{if 0.34 <}\boldsymbol{A}_{\boldsymbol{adj}}^{\boldsymbol{2}}\boldsymbol{\leq0.60} \end{aligned} \\ \exp\left( \boldsymbol{1.2937-5.709}\boldsymbol{A}_{\boldsymbol{adj}}^{\boldsymbol{2}}\boldsymbol{+0.0186}{\boldsymbol{(A}_{\boldsymbol{adj}}^{\boldsymbol{2}}\boldsymbol{)}}^{\boldsymbol{2}} \right)\boldsymbol{if 0.60 <}\boldsymbol{A}_{\boldsymbol{adj}}^{\boldsymbol{2}}\boldsymbol{\leq13.467} \\ \boldsymbol{0 if}\boldsymbol{A}_{\boldsymbol{adj}}^{\boldsymbol{2}}\boldsymbol{>13.467} \end{aligned} \right.$ (3)

## Microscopy analysis of biofilm viability

Biofilms grown on sterile coverslips were washed with filter-sterilized water to remove unbound bacteria. Subsequently, biofilms were stained for fluorescence with a biofilm viability kit (FilmTracer^TM^ LIVE/DEAD, Molecular Probes, Life Technologies Ltd.) according to the instructions provided by the manufacturer to observe the viability of bacterial cells inside the biofilm **(Supplementary Figure 4).** The LIVE/DEAD biofilm viability kit provided a visual representation of live and dead bacterial cells within the biofilm by green or red staining, respectively, based on the membrane integrity of the bacterial cells inside the biofilm. The kit contains two fluorescent nucleic acid stains: SYTO 9 and propidium iodide (PI). Briefly, a working solution of fluorescent stains was prepared by adding 3 µL of SYTO 9 stain and 3 µL of PI stain to 1 mL of filter-sterilized water. A 100 µL volume of staining solution was applied onto the biofilm and after 15 min incubation at room temperature in the dark, samples were rinsed with filter-sterilized water to remove the excess dyes from the stained samples.

Stained biofilms were examined by a confocal laser scanning microscope (CLSM) system (Leica TCS SP8 CLSM, Leica Microsystems Heidelberg GmbH, Manheim, Germany) with a 63x oil immersion objective lens with a numerical aperture of 1.4 (HC SPO CS2, Leica). During CLSM imaging, SYTO 9 emits green fluorescence and detects live bacteria with intact cell membranes, whereas propidium iodide (PI) emits red fluorescence and stains dead bacteria with damaged cell membranes. SYTO 9 dye was excited at 488 nm and the emission was measured from 500–550 nm; PI dye was excited at 561 nm and the emission was measured from 675–750 nm. Scanning speed was 600 lines/sec.  Images were produced from the microscope data using commercial software (LAS X, Leica Microsystems), and red-green 2D and 3D intensity maps were generated from the pixel data with ImageJ software and COMSTAT. For 3D reconstructions, 54 images were taken in each stack, with a z-step size of 0.4 μm, as CLSM has limited imaging depth. The percentage of viable and dead bacteria in each image was determined from the CLSM images using an open-source biofilm viability checker tool (Fiji macro (Mountcastle et al. 2021)).

## Agar Raman spectroscopy and OCT optical properties

Brain heart infusion (BHI) agar is a nutrient rich media that could contribute to both the RS and OCT signals as background noise. To this end, RS measurements of BHI agar plates and BHI supplemented with hemin and NAD (sBHI) agar plates were acquired under the same parameters (i.e., power = 24 mW, 15 s integration time, 100x objective) as the bacterial colonies on agar measurements. Under these conditions, the signal intensities of both agars were minimum, and there were no overlapping Raman peaks with those identified by the SMLR model as key peaks for bacterial colony differentiation (weighted features >60%), as shown in **Supplementary Figure 5**.

OCT images were also taken of BHI agar alone under similar measurement parameters as the bacterial colonies. From these images, the mean RI of BHI agar is 1.42 and the mean RI values of bacterial colonies are 1.43 – 1.45. In addition, the mean AC of BHI agar is minimal (0.52 mm^- 1^) compared to mean AC values from the bacterial colonies (2.91 – 4.25 mm^-1^).

# Supplementary Figures and Tables

## Supplementary Figures


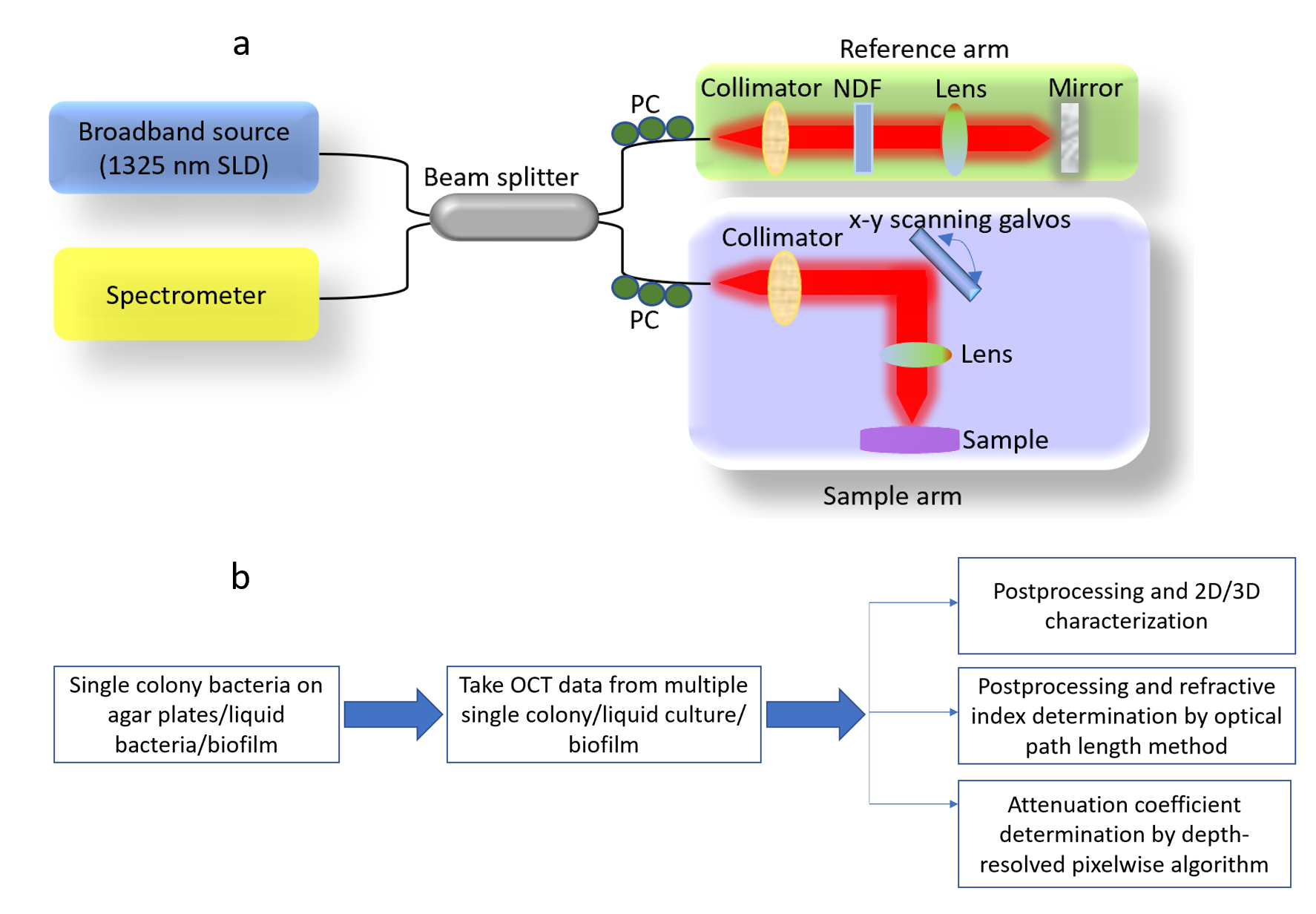
 **Supplementary Figure 1**. (a) Schematic of the spectral domain OCT system. PC = Polarization controller, NDF = Neutral density filter. (b) Workflow diagram for OCT imaging system.

**
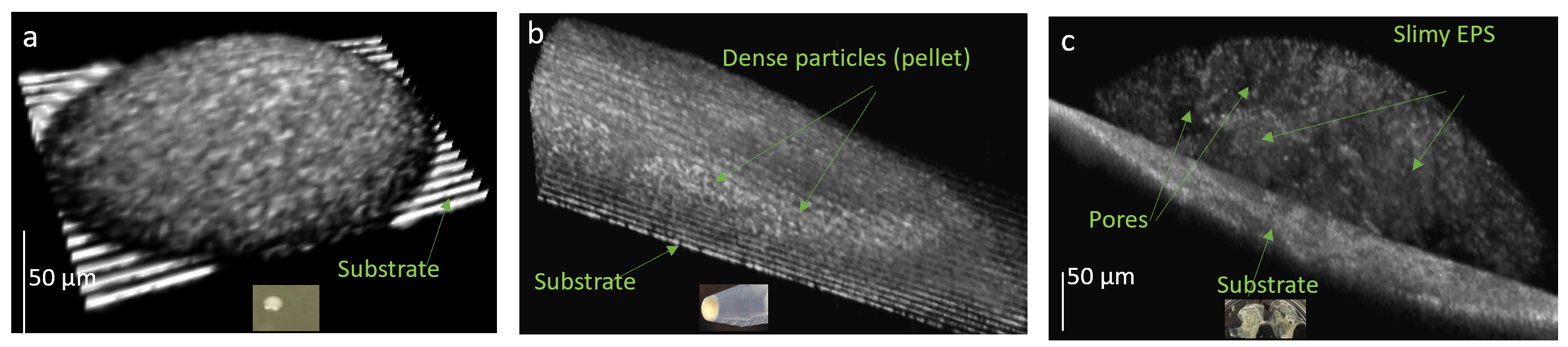
**

**Supplementary Figure 2.** Representative 3D OCT images from *P. aeruginosa* are shown in (a) a single-colony, (b) planktonic (in pellet) and (c) biofilm.

*
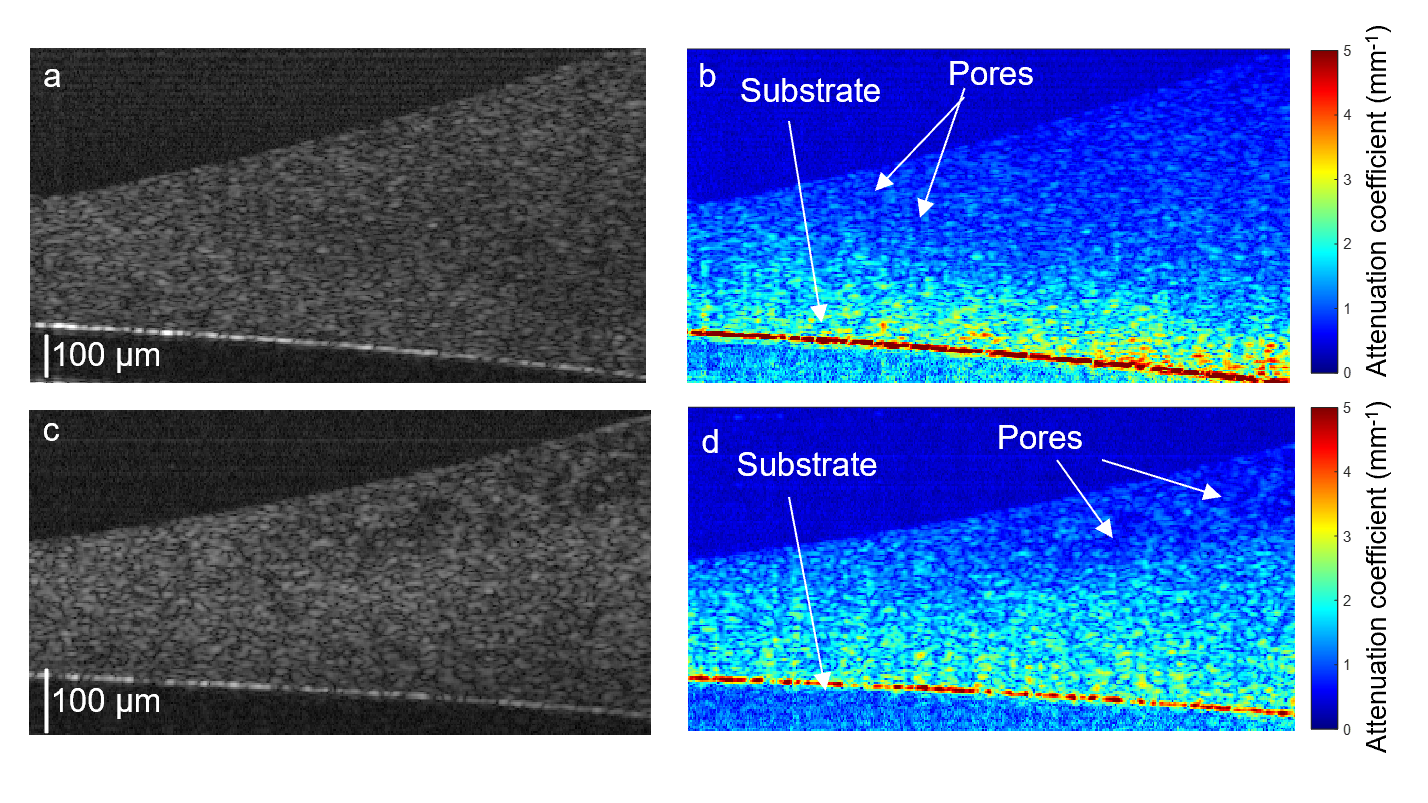
*

**Supplementary Figure 3.** Cross-sectional OCT images from representative (a) *S. pneumoniae* biofilm and (c) *M. catarrhalis* biofilm. Corresponding attenuation coefficient maps of a (b) *S. pneumoniae* biofilm and (d) *M. catarrhalis* biofilm.


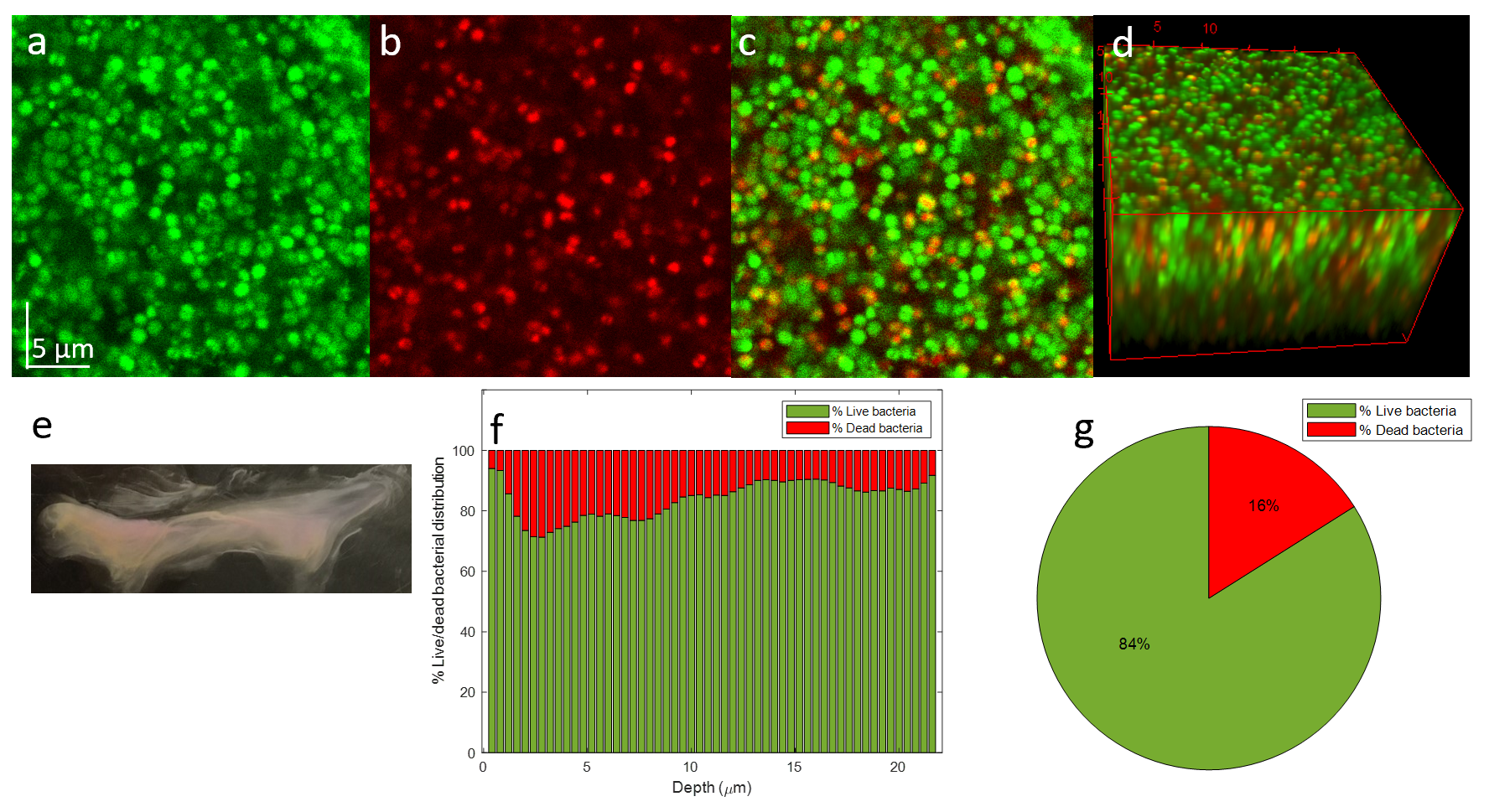


**Supplementary Figure 4**. CLSM images of bacteria viability inside a biofilm using a Live/Dead biofilm viability kit. (a) All bacteria cells stained with SYTO 9 (green fluorescent dye) are shown in the green channel. (b) Dead bacteria cells stained with PI (red dye) are shown in the red channel. (c) Merged image of both channels (Green – live cells, red – dead cells). (d) 3D reconstruction of bacterial viability in a biofilm (approximately 22 µm depth). (e) Stained biofilm on a coverslip. (f) Distribution of bacterial viability across the depth (up to 22 µm) of the biofilm. (g) Bacterial viability distribution in measured biofilm volume.

**

**

**Supplementary Figure 5**. Averaged Raman spectra highlighting key Raman features identified by SMLR to differentiate OM-causing bacterial colonies grown on BHI and sBHI agar. Red bands indicate features with SMLR feature weights above the threshold of ≥60%.

## Supplementary Table

**Table S1.** The A^2^­_adj_ test statistics, *p*-values and normality test results of refractive index (RI) and attenuation coefficient (AC) data for OM-causing bacterial species in all three forms.

| (n = 32). | Normality test of RI data | | | Normality test of AC data | | |
| --- | --- | --- | --- | --- | --- | --- |
|  | A^2^­_adj_ | *p*-value | Normality  (1: Normal if  *p* > 0.05;  0: Not normal, otherwise) | A^2^­_adj_ | *p*-value | Normality  (1: Normal if  *p* > 0.05;  0: Not normal, otherwise) |
| Colony |  |  |  |  |  |  |
| *H. influenzae* | 0.412 | 0.320 | 1 | 0.540 | 0.153 | 1 |
| *S. pneumoniae* | 0.368 | 0.408 | 1 | 0.640 | 0.087 | 1 |
| *M. catarrhalis* | 0.702 | 0.060 | 1 | 0.310 | 0.530 | 1 |
| *P. aeruginosa* | 0.471 | 0.229 | 1 | 0.440 | 0.280 | 1 |
| Planktonic (pellet) | | | | | | |
| *H. influenzae* | 0.644 | 0.085 | 1 | 0.510 | 0.187 | 1 |
| *S. pneumoniae* | 0.425 | 0.299 | 1 | 0.650 | 0.080 | 1 |
| *M. catarrhalis* | 0.530 | 0.162 | 1 | 0.410 | 0.317 | 1 |
| *P. aeruginosa* | 0.704 | 0.060 | 1 | 0.520 | 0.172 | 1 |
| Biofilm | | | | | | |
| *H. influenzae* | 0.440 | 0.275 | 1 | 0.620 | 0.100 | 1 |
| *S. pneumoniae* | 0.399 | 0.345 | 1 | 0.390 | 0.366 | 1 |
| *M. catarrhalis* | 0.688 | 0.066 | 1 | 0.460 | 0.240 | 1 |
| *P. aeruginosa* | 0.382 | 0.380 | 1 | 0.590 | 0.114 | 1 |

# Reference

Mountcastle, S. E., Vyas, N., Villapun, V. M., Cox, S. C., Jabbari, S., Sammons, R. L., Shelton, R. M., Walmsley, A. D., and Kuehne, S. A. (2021). Biofilm viability checker: An open-source tool for automated biofilm viability analysis from confocal microscopy images. *NPJ Biofilms and Microbiomes* 7 (1), 1-12. doi: 10.1038/s41522-021-00214-7.
